# Improving difficult peripheral intravenous access requires thought, training and technology (DART^3^): a stepped-wedge, cluster randomised controlled trial protocol

**DOI:** 10.1186/s12913-023-09499-0

**Published:** 2023-06-07

**Authors:** Jessica A Schults, Nicole Marsh, Amanda J Ullman, Tricia M Kleidon, Robert S Ware, Joshua Byrnes, Emily Young, Lisa Hall, Gerben Keijzers, Louise Cullen, Pauline Calleja, Steven McTaggart, Nathan Peters, Stuart Watkins, Amanda Corley, Christine Brown, Zhen Lin, Frances Williamson, Luke Burgess, Fiona Macfarlane, Marie Cooke, Callan Battley, Claire M Rickard

**Affiliations:** 1 The School of Nursing, Midwifery and Social Work, The University of Queensland, Brisbane, Australia; 2 Centre for Clinical Research, The University of Queensland, Brisbane, Australia; 3 Herston Infectious Diseases Institute, Metro North Health, Brisbane, Australia; 4 Nursing Midwifery Research Centre, Royal Brisbane and Women’s Hospital, Brisbane, Australia; 5 School of Nursing and Midwifery, Alliance for Vascular Access Teaching and Research, Griffith University, Queensland, Australia; 6 Children’s Health Queensland Hospital and Health Service, Brisbane, Australia; 7 Children’s Health Research Centre, The University of Queensland, Brisbane, Australia; 8 School of Medicine and Dentistry, and Menzies Health Institute Queensland, Griffith University, Southport, QLD Australia; 9 Centre for Applied Health Economics, Griffith University, Brisbane, QLD Australia; 10 School of Public Health, The University of Queensland, Brisbane, Australia; 11 Department of Emergency Medicine, Gold Coast University Hospital Southport, Queensland, Australia; 12 Faculty of Health Sciences and Medicine, Bond University, Gold Coast, Queensland, Australia; 13 Emergency and Trauma Centre, Royal Brisbane and Women’s Hospital, Brisbane, Australia; 14 School of Nursing, Midwifery & Social Science, Central Queensland University, Queensland, Australia; 15 Faculty of Medicine, University of Queensland, Queensland, Australia; 16 Department of Anaesthesia and Perioperative Medicine, Royal Brisbane and Women’s Hospital, Brisbane, Australia; 17 Jamieson Trauma Institute, Herston, QLD Australia

**Keywords:** Ultrasonography, Interventional, Catheterisation, Peripheral, Vascular access devices, Randomised controlled trial, Implementation science

## Abstract

**Background:**

Peripheral intravenous catheters (PIVCs) are the most used invasive medical device in healthcare. Yet around half of insertion attempts are unsuccessful leading to delayed medical treatments and patient discomfort of harm. Ultrasound-guided PIVC (USGPIVC) insertion is an evidence-based intervention shown to improve insertion success especially in patients with Difficult IntraVenous Access (BMC Health Serv Res 22:220, 2022), however the implementation in some healthcare settings remains suboptimal. This study aims to co-design interventions that optimise ultrasound guided PIVC insertion in patients with DIVA, implement and evaluate these initiatives and develop scale up activities.

**Methods:**

A stepped-wedge cluster randomized controlled trial will be conducted in three hospitals (two adult, one paediatric) in Queensland, Australia. The intervention will be rolled out across 12 distinct clusters (four per hospital). Intervention development will be guided by Michie’s Behavior Change Wheel with the aim to increase local staff capability, opportunity, and motivation for appropriate, sustainable adoption of USGPIVC insertion. Eligible clusters include all wards or departments where > 10 PIVCs/week are typically inserted. All clusters will commence in the control (baseline) phase, then, one cluster per hospital will step up every two months, as feasible, to the implementation phase, where the intervention will be rolled out. Implementation strategies are tailored for each hospital by local investigators and advisory groups, through context assessments, staff surveys, and stakeholder interviews and informed by extensive consumer interviews and consultation. Outcome measures align with the RE-AIM framework including clinical-effectiveness outcomes (e.g., first-time PIVC insertion success for DIVA patients [primary outcome], number of insertion attempts); implementation outcomes (e.g., intervention fidelity, readiness assessment) and cost effectiveness outcomes. The Consolidated Framework for Implementation Research framework will be used to report the intervention as it was implemented; how people participated in and responded to the intervention; contextual influences and how the theory underpinning the intervention was realised and delivered at each site. A sustainability assessment will be undertaken at three- and six-months post intervention.

**Discussion:**

Study findings will help define systematic solutions to implement DIVA identification and escalation tools aiming to address consumer dissatisfaction with current PIVC insertion practices. Such actionable knowledge is critical for implementation of scale-up activities.

**Trial registration:**

Prospectively registered (Australian and New Zealand Clinical Trials Registry; ACTRN12621001497897).

**Supplementary Information:**

The online version contains supplementary material available at 10.1186/s12913-023-09499-0.

## Background

Peripheral intravenous catheters (PIVCs) are the most common invasive medical device [2], but can be difficult to insert. Up to 67% of first insertion attempts are unsuccessful, and 10–45% of patients require upwards of three insertion attempts [3, 4]. Multiple PIVC insertions are painful and an unnecessary burden on the health system through increased clinical workloads and cost [5]. One in three patients presenting to an emergency department, and one in two hospitalised patients present with Difficult IntraVenous Access (DIVA), making this a global issue that impacts millions annually [3, 6, 7]. This rate is likely to increase in coming years with growing chronic disease and morbidity burden in the community [8]. DIVA has various definitions [1, 9] but generally includes few or limited visible and/or palpable suitable veins, since these are the main criteria traditionally used to guide insertion using the “landmark” technique [10, 11].

Landmark PIVC insertion technique is typically used for PIVC insertion. However, the technique has limitations. It does not allow for the comprehensive assessment of vein caliber, depth, valve location, or tortuosity before device insertion, further tip position within vessel is unable to be confirmed (rather than the tissue) post-procedure [12]. An alternative to landmark insertion is ultrasound-guided (USG) insertion. This approach is well established for central venous catheters insertion but used less frequently for PIVC insertions [13]. Ultrasound provides the opportunity for thorough pre-assessment of the vein and enables visualisation of the needle and cannula throughout the entire PIVC insertion procedure [11, 12]. Over the past decade portable ultrasound machines have become increasingly available for use in practice - with excellent resolution for viewing peripheral veins [14]. Compared to landmark technique, USG insertions require fewer insertion attempts, less insertion time, and increase patient satisfaction [12, 15]. With a recent systematic review (8 studies; *n* = 1660 patients) confirmed overall superiority for USG insertion over landmark technique for PIVC insertion success (regardless of DIVA status; 81% vs. 70% respectively; odds ratio [OR] 2.49, 95% CI 1.37–4.52 [12]).

Currently, the choice of insertion technique (landmark or USG) by individual practitioners is inherently influenced by inserter experience and whether ultrasound training and equipment is available. The assessment of a patient’s DIVA status is inconsistently performed in practice [16]. To date, there is minimal evidence on the clinical impact of DIVA identification tools or escalation pathways (to more skilled inserters with USG skills) on patient outcomes [9, 16]. Consumers who have experienced DIVA report PIVC insertion practices vary widely across healthcare institutions influencing their experience and satisfaction with care [16, 17]. For patients who experience DIVA, ample evidence exists to support ultrasound-guided insertion as a first approach, rather than a rescue tool following failed landmark insertions [6, 18–20]. There is no known adverse effect of using ultrasound for PIVC insertion, and as such ultrasound-guided insertion is recommended for patients who experience in DIVA in international practice guidelines and clinical standards [21–23]. Despite its recognised efficacy and value, the implementation of ultrasound-guided PVC insertion is limited in many healthcare institutions, and current workforce and systems require purposeful adaptation [2, 9, 24]. This limitation is largely due to the requirement of additional equipment and processes, staff training and skills assessment. To achieve substantial patient and system benefit, prospective identification and escalation of patients with DIVA is required [3].

## Methods / design

### Study aims

The overall aim of the Difficult Access Requires Thought, Training and Technology (DART^3^) study is to implement and evaluate effective DIVA identification and escalation pathways to support PIVC insertion in adults and children, including appropriate use of ultrasound-guided procedures. The specific objectives of the study include the following:

1. To develop hospital-based interventions that support DIVA identification and escalation, adapted to the local health care context.
2. To implement co-designed DIVA identification and escalation pathways, and evaluate their clinical effectiveness, cost effectiveness and implementation strategies.
3. Develop recommendations for future strategic options for scale-up and optimised implementation of DIVA identification and escalation pathways in different settings.

### Hypotheses

The primary hypothesis is that implementation of tailored, co-designed DIVA identification and escalation pathways will significantly increase the incidence of first attempt PIVC insertion success for patients identified as having DIVA (primary outcome).

#### Secondary hypotheses

That implementation of the DIVA identification and escalation pathways:

H_2_ Will significantly increase the incidence of first attempt PIVC insertion success for **all patients**.

H_3_ Increases the proportion of patients with DIVA who have ultrasound used at first, or any attempt.

H_4_ Reduces the number of PIVC insertion attempts.

H_5_ Results in higher rates of successful PIVC placement and shorter time-to-therapy.

H_6_ Results in higher rates of alternate device or route use.

H_7_ Reduces post-insertion PIVC failure and complications.

H_8_ Results in increased PIVC dwell time.

H_9_ Is sustainable (i.e. There is no significant reduction in first time insertion success at 3 or 6 months compared to full implementation (month 10).

H_10_ Reduces rates of unnecessary PIVCs.

H^11^ Is cost-effective in Queensland healthcare settings.

H^12^ Reduces rates of cluster level routinely collected rates of primary BSI and S. Aureus BSI.

H^13^ Improves patient/carer and staff satisfaction with insertion procedure.

### Study design

**Fig. 1:**
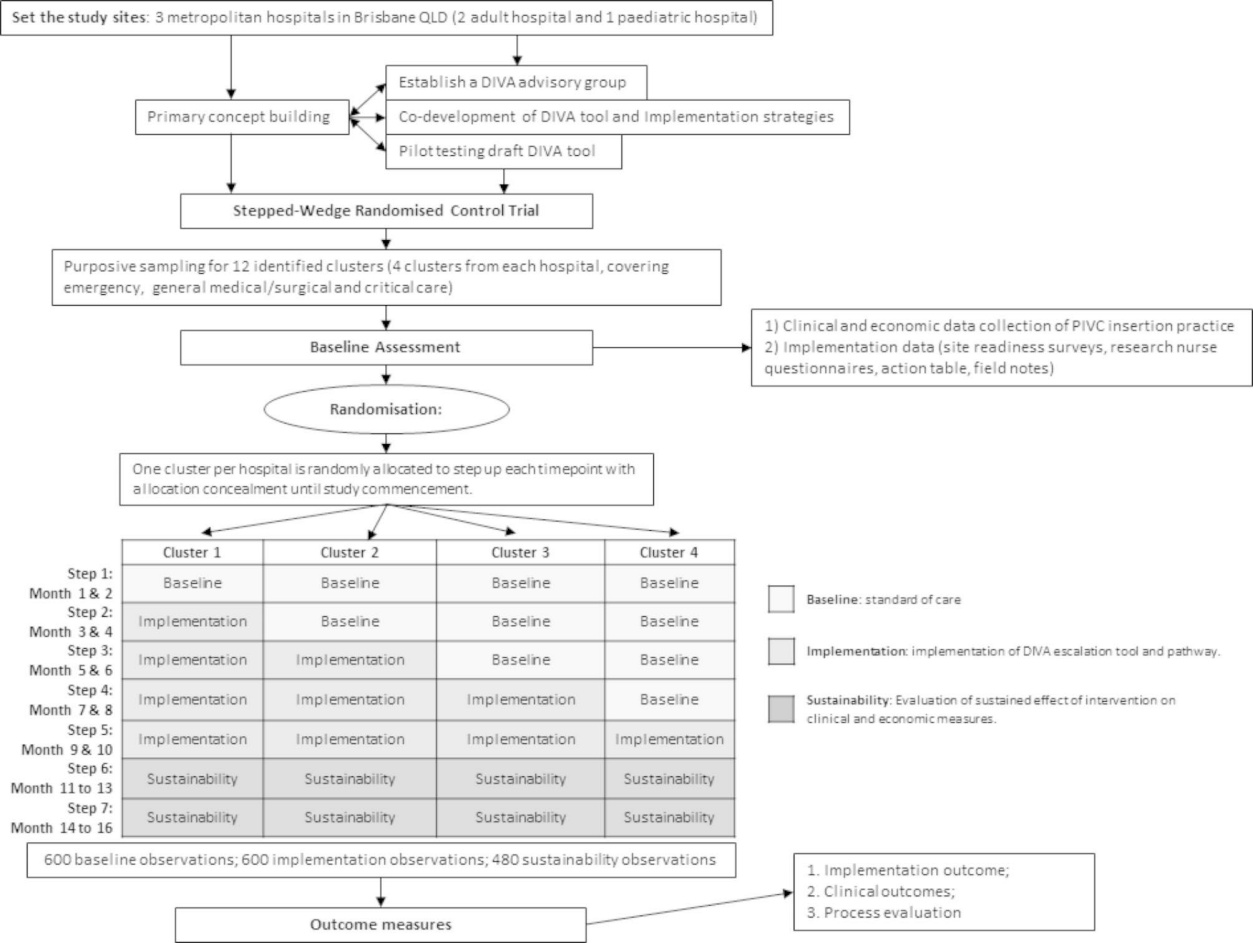
Study flow diagram

**Fig. 2:**
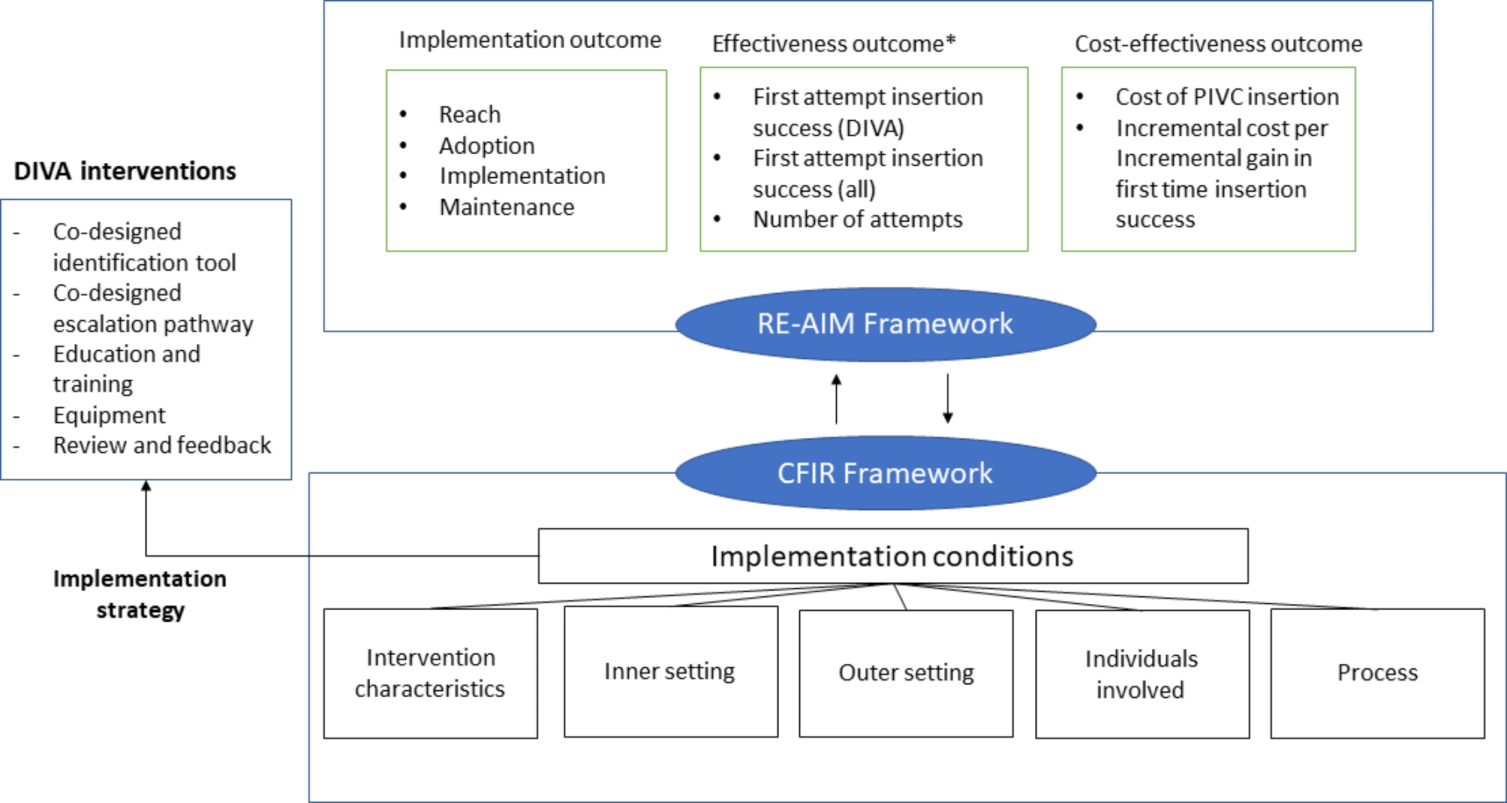
Theoretical model for DART^3^ study *Examples of clinical effectiveness outcomes, not all outcomes included

A stepped wedge cluster randomised trial (SW-CRT) will be used to evaluate the implementation and effectiveness of co-designed DIVA identification and escalation pathways (Fig. 1). The theoretical model for DART3 is outlined in Fig. 2. The RE-AIM (Reach, Effectiveness, Adoption, Implementation, and Maintenance) framework [25, 26] will be used to evaluate the strategy, in which three domains of outcomes will be included: effectiveness outcomes, cost-effectiveness outcomes and implementation outcomes. Effectiveness will be measured using health service and patient outcomes. Implementation evaluation will explore reach, adoption, implementation, and maintenance. The Consolidated Framework for Implementation Research [27] will be used to identify the barriers and facilitators influencing the implementation strategy and to establish the feasibility, acceptability, and sustainability of our DIVA initiatives. The study will be reported in line with Standard Protocol Items: Recommendations for Interventional Trails (SPIRIT) [28]. The completed SPIRIT checklist is provided in supplementary material 7.

### Setting and sampling

DART^3^ is a multi-site study involving three large tertiary hospitals in South-East Queensland, Australia. All hospitals (two adult and one paediatric) are government-funded, university affiliated teaching facilities.

Each hospital has four participating clusters (wards or departments), for a total of 12 clusters. Each cluster begins with a baseline phase, followed by a step up to the implementation phase, where the intervention is introduced, the study is completed by a sustainability phase, divided into three- and six-month time points. The total study timeframe will be 16-months and consist of seven steps. Hospital and cluster characteristics are outlined in Supplementary material 1. Eligible clusters include emergency departments, inpatient wards or critical care units (CCU, ICU) where greater than 10 PIVCs per week are typically inserted. Operating services, which theatres, radiology, rehabilitation, or psychiatric units, were not eligible for inclusion. All hospitalised patients (DIVA or non-DIVA) of any age prescribed a PIVC insertion (standard or long length PIVC) may be included. Patients requiring time-critical intravenous access (e.g., Medical Emergency calls, intraosseous access) will not be included in the study.

### Intervention description

During the implementation phase, clusters not stepping up and non-participating wards will continue with usual care for PIVC insertion. The DART^3^ intervention involves a co-designed DIVA identification tool (clinical decision-making tool which stratifies the patients’ risk of having DIVA) and escalation pathway tailored to the local health service [1]. All concomitant cares will continue while the patient is enrolled in the trial. PIVC maintenance and removal procedures will be as per local hospital policies and driven by treating clinicians. Once the study PIVC is removed, as decided by the treating clinical team, outcome and adverse event data will be collected for a further 48-hours by clinical research nurses.

### Randomisation and blinding

The intervention will be applied at the cluster (ward) level. Intervention clusters will carry out the DIVA interventions, and clusters that have not been enrolled in the intervention will continue with usual care. Usual care will vary by site and cluster, (predominantly landmark insertion, with ad hoc DIVA assessment and/or escalation to a small number of ultrasound skilled practitioners, typically after multiple failed landmark attempts).

Randomisation to the sequence of implementation occurs at site level and is stratified by hospital, with each cluster a unit of randomisation. Cluster sequence generation is conducted by a centralised, web-based randomisation service (https://randomisation.griffith.edu.au/) to ensure concealment.

All clusters commence in the baseline phase and then in randomly allocated order, one cluster per site steps up to implementation of the intervention, approximately every two months as feasible for the site (stepped-wedge cluster-randomisation schema outlined in Supplementary material 2). Cluster allocations will be revealed to the project manager who will advise the sites on commencement of the baseline phase. This is necessary to allow time to plan cluster implementation strategies. Blinding of patients, clinicians and research staff is not possible due to the nature of the intervention. Outcome assessors apportioning infection outcomes (infectious diseases physician) will be blinded to study phase (baseline or implementation).

### Sample size

Approximately 40% of studied patients are expected to be DIVA patients (8 per cluster step), and 60% non-DIVA (12 per cluster step). For DIVA patients, we assume baseline first attempt insertion success of 50% (based on meta-analysis [12] and our unpublished data on file) and anticipate a 50% relative improvement to 75% first insertion success [12]. We assume baseline first insertion success in non-DIVA patients of 70%, rising to 80% post- intervention. This is equivalent to an overall change for all patients from 62 to 78% first attempt insertion success. With 240 patients at each of the five data collection periods (baseline plus four steps), there will be > 90% power to detect a significant difference between the usual care and implementation phases (alpha = 0.05). See Supplementary Material 3: statistical analysis plan.

To measure sustainability of the effect of the implementation on the primary endpoint, we will additionally assess 240 patients at each sustainability time point (3 and 6 months). This leads to a total sample size of 1680 observations, consisting of 240 observations at each of the 7 periods (baseline, steps 1–4, 3- and 6-month follow-up).

### Implementation approaches

#### Site-specific advisory groups and co-design of DIVA tools and implementation strategies

Advisory groups have and will be established at each hospital comprising of cluster and hospital level clinicians, vascular access experts, nurses and medical managers and educators, quality and safety experts, consumers, and hospital executives. To guide the co-design process, core concepts (e.g., patient risk factors) will be provided by the investigator team as important foundational principles of the tools [1, 17, 24]. These are based on a series of published preliminary work by the investigating team including evidence syntheses [9, 29, 30] and interviews (face-to-face and by telephone) [16, 17] conducted with key stakeholders across hospital and health services. Workshops and teleconference meetings with hospital groups and executives will be used to co-design tools tailored to the local context.

Site-specific advisory groups will also tailor multi-modal implementation strategies that are feasible for hospitals to support capability, opportunity, and motivation to use the DIVA tool and escalation pathway. Implementation strategies (outlined in supplementary material 4) will be underpinned by Michie et al. Behaviour Change Wheel implementation theory [31] with discussions informed by an implementation scientist (LH).

Site advisory group meetings, comprising stakeholders and champions, will continue throughout the study to tailor implementation strategies to ensure they are locally effective. Research nurses (majority skilled ultrasound PIVC inserters), supported by an overall study manager and research assistant will work with investigators, local educators, and clinical managers to deliver the implementation strategies. By the end of implementation, PIVC inserters will be the local accredited workforce, however research nurses will initially undertake bedside teaching and coaching as local inserters learn the ultrasound technique.

#### Pilot of co-designed tools

The draft DIVA identification pathway tool will be pilot tested at the partner hospitals to assess validity and reliability, and to refine for clarity and utility. Face and content validity of the DIVA identification pathway will be assessed using an electronic questionnaire sent via email to the advisory group. Item validity will be determined using a content validity index (CVI). A panel of experts (n ≥ 5 at each site) comprising multidisciplinary vascular access specialists will be asked to provide feedback on the appropriateness and relevance of tool domains/items using a four-point level of agreement (1, not; 2, somewhat; 3, quite; 4, highly) and face validity of the tool. These clinicians will be sourced from the clinical departments of partner hospitals, separate from the advisory group. Item level CVIs will be calculated as the number of experts giving a score of 3 or 4 (item cut off score of 0.75). Face validity tests the appearance of the DIVA identification tool is adequate for its intended purpose. Reproducibility of the DIVA identification tool will be assessed using inter-rater agreement by two PIVC inserters in 100 patients (200 paired assessments).

### Evaluation of the DART^3^ intervention

#### Outcome measures and data collection

Table 1 summarises DART^3^ outcome measures and data collection methods categorised by the RE-AIM framework. To measure outcomes, we will collect cluster and hospital level data [42], conduct surveys of healthcare providers and investigators, collect field notes and conduct research nurse check-in surveys.

**Table 1:** DART^3^ outcomes. Outcomes, definitions, and data collection organised by RE-AIM domains

Outcomes	Information	Data source
Patient and service level outcomes		
***Primary***		
First attempt insertion success in patients identified as DIVA	One needle puncture, by one inserter, to achieve successful insertion of a functional (can be aspirated/flushed) PIVC ^2^	Hospital-based assessments
***Secondary***		
First attempt insertion success for all patients (regardless of DIVA status)	One needle puncture, by one inserter, to achieve successful of a functional (can be aspirated/flushed) PIVC ^2^	Hospital-based assessments
Number of attempts	Number of skin punctures to attempt PIVC insertion ^3^	Hospital-based assessments
Procedure outcome:	Successful PIVC insertion; time from PIVC referral to PIVC insertion (censored at 48 h); alternate device; alternate route (e.g., oral) ^4^	Hospital-based assessments
PIVC failure	Composite measure of local infection, primary bloodstream infection (BSI), occlusion, infiltration/extravasation, dislodgement (includes leaking), thrombosis and/or phlebitis ^6 7^	Hospital-based assessments
Insertion/post-insertion complications	Bruising, haematoma, nerve injury, arterial puncture, or skin injury as well as the individual components of PIVC failure (above) ^6 8^	Hospital-based assessments
PIVC dwell time	Time from PIVC insertion to PIVC removal (in hours) ^6^	Hospital-based assessments
PIVC necessity	PIVC used for fluids or medications within 24 h (excluding patients who require a prophylactic PIVC in situ as part of their treatment e.g., status epilepticus) ^5^	Hospital-based assessments
Incidence of blood stream infection	Cluster level routinely-collected rates of primary BSI and S. Aureus BSI ^9^	Hospital-based assessments
Economic outcomes		
Cost-effectiveness	Direct and indirect healthcare costs to the health system, patients/carers: (time to insertion/therapy, cost of products, number of staff, staff time, costs of responding to failed insertion including cancelled appointments)	Hospital-based assessments
Implementation outcome- reach		
Number of healthcare professionals attending Ultrasound training	Counts of clinicians attending ultrasound training	Hospital-based assessments
Number of staff accredited in ultrasound insertion	Per local ultrasound accreditation requirements	Hospital-based assessments
Implementation outcome—adoption		
Healthcare provider engagement	Proportion of patients assessed using the DIVA vein assessment tool	Hospital-based assessments
Ultrasound adoption	proportion of DIVA patients with ultrasound used at the first, or any attempt	Hospital-based assessments
Patient/carer/parent satisfaction and pain with insertion procedure	0–10 numeric rating scale ^6^	Hospital-based assessments
Inserter (initial and/or successful inserter) satisfaction with escalation pathway	0–10 numeric rating scale ^6^	Hospital-based assessments
Attitude of healthcare providers with DIVA tools	Degree of acceptability of the DIVA tools by clinicians	Key stakeholder interviews
Implementation outcome - implementation		
Fidelity	Degree that the DIVA tools are implemented as planned in original protocol	Key stakeholder interviews
Feasibility	Extent that the DIVA tools can be carried out in specific settings	Key stakeholder interviews
Outer context	Macro-level external factors including social, funding, and leadership	Key stakeholder interviews
Inner context	Micro-level internal factors including behaviours, feedback.	Key stakeholder interviews
Implementation outcome—maintenance		
Sustainability of the intervention and effectiveness	First time insertion success at 3- or 6-month sustainability assessments	Hospital-based assessments
Satisfactory of stakeholders	0–10 numeric rating scale ^6^	Key stakeholder interviews
Financial sustainability	Explored in the cost-effectiveness analysis and qualitatively through interviews with executive stakeholders.	Key stakeholder interviews
Institutionalisation of interventions	Concerned with sustaining social behavioural change	Key stakeholder interviews

*RE-AIM* Reach, effectiveness, adoption, implementation, maintenance

Data collection will be facilitated using a custom-built REDCap (Research Electronic Data CAPture http://project-redcap.org/ Vanderbilt, USA) database. Research nurses will directly enter de-identified study data (data variables and time points outlined in Table 2 and supplementary material 5) in the clinical areas using study tablets with the REDCap application configured for this trial.

**Table 2:** Data collection time points for DART^3^

Activities	Pre-implementation	SWRCT - implementation						
		Step 1	Step 2	Step 3	Step 4	Step 5	Step 6	Step 7
Establish site-specific advisory groups	**√**							
Qualitative interviews and evidence synthesis	**√**							
Economic interviews for PIVC insertion procedures	**√**	**√**	**√**	**√**	**√**	**√**		
Co-develop DIVA tool and escalation pathway	**√**							
Pilot-test and evaluate DIVA tool and escalation pathway prototypes for reliability, acceptability and reproducibility	**√**	**√**						
Implementation of site-specific DIVA tools across the 3 hospitals, 12 clusters in a staggered manner			**√**	**√**	**√**	**√**		
Cluster 1 from each hospital			**√**	**√**	**√**	**√**		
Cluster 2 from each hospital				**√**	**√**	**√**		
Cluster 3 from each hospital					**√**	**√**		
Cluster 4 from each hospital						**√**		
Patient and service level outcomes (REDCap CRF)		**√**	**√**	**√**	**√**	**√**	**√**	**√**
DIVA PIVC time-in-motion data for cost-effectiveness analysis		**√**	**√**	**√**	**√**	**√**		
Implementation data collection (questionnaires, field notes)		**√**	**√**	**√**	**√**	**√**		
Sustainability activities							**√**	**√**
Organisational guideline incorporation							**√**	**√**
Process evaluation interview						**√**	**√**	**√**

##### Surveys of healthcare providers

We will conduct a series of informal short surveys with research staff over the course of the study to monitor progress. This will include.

- Monthly research nurse check-ins;
- Cluster readiness surveys (conducted once during baseline and monthly in the implementation period);
- Site investigator surveys (quarterly in implementation and monthly in the sustainability period).

Research nurses will compile field notes and complete an action table capturing intervention components over the study duration. This information will be collated and narratively summarised.

### Evaluation of DART^3^ implementation strategy and process evaluation

A mixed methods process evaluation will be undertaken incorporating quantitative and qualitative measures of intervention activities (such as number of participants and delivered components), and an exploration of the interaction between the intervention and the contextual characteristics of the three participating hospitals [32]. The primary aim of the process evaluation is to describe how the DART^3^ intervention functioned across different settings, including if and why it has different effects. The domains of focus for the process evaluation are outlined in Supplementary material 6 and include an exploration of:

1. How the intervention was implemented;
2. How people participated in, and responded to the intervention, including patients, over time;
3. The contextual characteristics (managerial, economic, organisational and work level) that facilitated this relationship; and.
4. How well the Behaviour Change Wheel (COM-B) theory underpinning the intervention was realised in the intervention design and implemented at each site.

Data collection methods will include interviews, descriptive field notes, regular surveys with research staff and informal conversations and meetings. Key stakeholder interviews willl take place with purposively sampled participants across clusters in the implementation and sustainability period. A semi-structure interview guide will be utilised and include three forced response questions to determine clinicians’ acceptability, satisfaction, and perception of tool suitability for its intended purpose (measured on a 11-point Likert scale).

Interview transcripts will be professionally transcribed and analysed using reflexive thematic analysis and mapped to CFIR domains [33–35]. This mapping will complement quantitative data collected to evaluate the implementation strategy and highlight emerging themes to identify barriers and facilitators contributing to implementation of the DART^3^ interventions. All other data will be aggregated to identify key themes across all data sources, sites, and participants — to identify variation of views, experiences and practices within each hospital site and cluster. This will include the development of schematic diagrams. The CFIR framework will be used to identify factors that influence intervention implementation and effectiveness [36, 37]. Data will be organised for analysis by CFIR domain (e.g., inner setting, outer setting) to make between-cluster/hospital comparisons and identify trends in how clusters/hospitals experienced implementation.

The process evaluation results will inform the development of scale up activities for DIVA tools through the identification of barriers and facilitators to program implementation, fidelity and feasibility [38]. Contextual factors associated with optimal implementation will be explored to inform optimisation and adaption of DIVA tools and future implementation strategies for patients with DIVA.

### Evaluation of DART^3^ primary and secondary intervention outcomes (Effectiveness evaluation)

#### Statistical methods

Patient variables, staff and consumer satisfaction ratings, will be summarised using descriptive statistics. Mean and standard deviation will be used for normally distributed data, and median and interquartile range for data not normally distributed. Counts and percentages will be used to summarise categorical data. The between-group comparison of the primary outcome will be analysed using a mixed-effects logistic regression model. Fixed effects included in the model will be study phase (pre/post implementation) and step. Cluster will be included as a random effect to account for probable non-independence of observations within clusters. The individual will be the unit of analysis. Continuous secondary outcomes will be compared with mixed-effects linear regression, time-to-event secondary outcomes by multilevel survival modelling, and count outcomes by mixed-effects Poisson regression. All models will account for clustering. The primary analysis will be ‘intention-to- treat’, with ‘per protocol’ analyses assessing the effect of protocol compliance. A statistical analysis plan is outlined in Supplementary material 3.

#### Economic evaluation

The primary cost-effectiveness analysis will be conducted from a health system perspective as informed by the time-and-motion sub-study. The primary outcome will be the incremental cost per incremental gain in first time insertion success. Results will be presented as incremental cost-effectiveness ratios (ICERs) with 95% credible intervals. ICER will be estimated based on the incremental costs and effect between data collected from patients in the baseline phase compared to data collected in the implementation phase. The developed DIVA identification and escalation pathways and implementation strategies will be cost-effective if the ICER ≤ a priori cost effectiveness threshold (λ) for the value of first-time insertion sourced from the literature. Costs associated with implementation will be annualised over assumed life expectancy of practice change (seven years) and attributed to each patient based on total target patient population estimates.

#### Time-and-motion sub-study

To inform the cost-effectiveness analysis, we will use a prospective, observational time-and-motion design to determine staff time and resource use for PIVC insertion procedures. This design is well-suited to healthcare settings with complex work processes and has been used previously to assess and improve hospital-based procedures [39, 40]. This sub-study was designed in line with the Suggested Time and Motion Procedures (STAMP) checklist [41]. PIVC insertions will be purposefully sampled across RCT clusters (wards/clinical units) and personnel skill level to account for any differences that these variables may have on outcomes. We will aim for a sample size of 27 DIVA insertions during baseline and again during implementation, to meet general central limit theorem requirements.

#### Data management

Data will be kept in locked premises, password protected, or in locked filing cabinets onsite. In keeping with the relevant policies regarding retention and disposal of clinical research records, the information will be retained for 15 years. This also fulfils the Australian Code for the Responsible Conduct of Research requirements for retention of research data. Missing data will be considered in analyses and reported in the results of the trial. The project manager and research assistant will undertake quality checks at each site to ensure allocation integrity, query missing or implausible data, and monitor 100% source data verification for: first patient studied in each cluster, primary outcomes, and a random 5% of other data for all patients.

Quality and completeness of the data will be monitored throughout the study by the project manager and research assistant, with regular study meetings to follow up on missing or incomplete data entries. Protocol non-adherence and missing data will be tracked and communicated to site investigators and research nurses on a regular basis to promote compliance and data completeness.

#### Confidentiality

Trial patients will not be individually identified in the presentation of results. All clinical details will be entered in coded format and the confidentiality of the data will be maintained unless disclosure is required by law. For participants involved in process evaluation interviews, all identified data will be deleted once interviews are deidentified for analysis. Participants will be differentiated using anonymous codes, and the confidentiality of the participant will be maintained unless disclosure is required by law.

### Oversight and monitoring

#### Composition of the coordinating centre and trial steering committee

The project coordinating centre consists of the principal and chief investigators, project manager and research assistants. The principal investigator meets with the project manager and research assistants frequently to review progress, discuss challenges, and address issues and concerns of trial delivery. Project staff, including project manager and research assistants, meet with site members regularly to discuss site progress, implementation strategies and compliance challenges. Study investigator meetings are held every quarter and provide regular progress updates to the broader project team.

#### Adverse event reporting and harms

This is a minimal risk, standard-of-care implementation trial. PIVC related adverse events which occur as part of standard PIVC insertion and maintenance include rare extravasation injuries and catheter-associated bloodstream infections. All adverse events will be reviewed by site investigators and a summary provided to the HREC as adverse events, during annual reporting.

### Dissemination, policy dialogue and national road trip

Dissemination will be undertaken using aggregated data only. Results will be presented locally, and at relevant national and international scientific meetings. The study will be published in an open-access peer-reviewed healthcare journal with authorship consistent with the International Committee of Medical Journal Editors’ criteria. Media releases will be circulated through appropriate professional bodies and organizations (e.g., The University of Queensland, The Australian Commission on Safety and Quality in Healthcare). We will translate the findings and results of this study into policy documents and reports. Reports will be distributed through university, health service partner and investigator networks to relevant decision-making bodies and agencies. We will place resources supporting further dissemination on a purpose-built DIVA ultrasound webpage with downloadable materials including: published findings; consumer and health professional fact sheets; educational materials; DIVA identification tools and decision-support pathways; successful implementation strategies; economic modelling; and workforce options.

## Discussion

Clinical practice guidelines cite the supporting evidence and recommend USG PIVC insertion for DIVA patients [21–23], however, widespread implementation of these guidelines in many hospitals has been slow [2, 16, 17]. This may be due to the lack of implementation resources to identify, coordinate and operationalise use of USGPIVC insertion in healthcare settings. As such there is an urgent need for new implementation strategies to support better PIVC insertion practices in all patients, but specifically in patients who experience DIVA. This project will be the first to develop and implement hospital-based interventions in Australia to improve the quality-of-care of people who experience DIVA.

The DART^3^ trial will inform the uptake of USG PIVC insertion practice for patients with DIVA. This study will observe clinicians practicing directly with the study interventions in their clinical environments, identifying practical barriers and supports for the implementation of DIVA pathways and USG PIVC insertion into clinical care both in and out of hours. The broad range of settings in this study will provide rich contextual data to inform sustainable and scalable implementation of site-specific DIVA identification tools and escalation pathways to support USGPIVC insertion within healthcare settings.

However, this is a real-world pragmatic stepped wedge trial, there are concerns related to confounding, bias, and temporal trends that may limit the validity of the findings. The pragmatic design was chosen because it allows iterative feedback of results internally, to support study improvements and engagement; it is anticipated that the project intervention will be well received across all participating hospitals. Nevertheless, we will use a modest effect size, cluster randomisation, and an analysis plan to mitigate these limitations. Second, the voluntary participation of cluster participants limits the generalisation of our results. Willingness of participating clinicians in clusters to participate in the DART^3^ interventions may indicate a greater focus on quality improvements related to intravenous catheter insertion and thus may limit reproducibility at other hospitals. Nevertheless, we will attempt to lessen these limitations at baseline through randomisation.

## Trial status

The trial is registered with Australian New Zealand Clinical Trials Registry (ANZCTR), ACTRN12621001497897. The SWCRT is currently recruiting participants and is anticipated to reach completion around July 31st 2023.

## Protocol

Version 1.0; Date: 19th May 2021.

## Electronic supplementary material

Below is the link to the electronic supplementary material.

Supplementary Material 1

Supplementary Material 2

Supplementary Material 3

Supplementary Material 4

Supplementary Material 5

Supplementary Material 6

Supplementary Material 7

## Data Availability

Data may be available for collaborators on request to the CI, Professor Rickard (c.rickard@uq.edu.au).

## Abbreviations

DART3: Difficult intravenous access requires thought, training and technology
DIVA: Difficult intravenous access
HREC: Human research and ethics committee
ICER: Incremental cost effectiveness ratios
IV: Intravenous
NHMRC: National Health and Medical Research Council
PIVC: Peripheral intravenous catheter
RCT: Randomized controlled trial
ReN: Research nurse
SUR: Seemingly Unrelated Regression
SW-CRT: Stepped wedge cluster randomized trial
STAMP: Suggested Time and Motion Procedures
USG PIVC: Ultrasound-guided peripheral intravenous catheter
VAD: Vascular access device
REDCap: Research Electronic Data CAPture
