# Supplementary material for: Improving difficult peripheral intravenous access requires thought, training and technology (DART3): a stepped-wedge, cluster randomised controlled trial protocol
Source: BMC Health Serv Res. 2023 Jun 7;23:587. doi: 10.1186/s12913-023-09499-0 (PMC10249237; doi:10.1186/s12913-023-09499-0)
Supplement: Supplementary file 1 — Supplementary Material 1 [file 12913_2023_9499_MOESM1_ESM.docx]

**Supplementary material 1. Characteristics of DART^3^ hospitals**

| **Hospital and location** | **Setting** | **Inpatient Beds** | **Cluster wards** |
| --- | --- | --- | --- |
| Gold Coast University Hospital; Gold Coast, QLD | Metropolitan | 750 | - Emergency/Medical Decision Unit - Cancer and Blood Disorders - Vascular - Trauma and Orthopaedics |
| Queensland Children’s Hospital; Brisbane, QLD | Metropolitan | 359 | - Paediatric Intensive Care - Inpatient Surgical - Inpatient Medical - Infants including neonates |
| Royal Brisbane and Women’s Hospital; Brisbane, QLD | Metropolitan | 929 | - General Surgical - General Surgical and Colorectal - Coronary Care Unit - Emergency and Trauma Centre |

QLD = Queensland
