# Supplementary material for: Improving difficult peripheral intravenous access requires thought, training and technology (DART3): a stepped-wedge, cluster randomised controlled trial protocol
Source: BMC Health Serv Res. 2023 Jun 7;23:587. doi: 10.1186/s12913-023-09499-0 (PMC10249237; doi:10.1186/s12913-023-09499-0)
Supplement: Supplementary file 2 — Supplementary Material 2 [file 12913_2023_9499_MOESM2_ESM.docx]

| **Hospital/Dept.** | **Baseline** | **Implementation** | | | | **Sustainability** | | **Total** |
| --- | --- | --- | --- | --- | --- | --- | --- | --- |
|  |  | Step 1 | Step 2 | Step 3 | Step 4 |  |  |  |
| Month | 1-2 | 3-4 | 5-6 | 7-8 | 9-10 | +3mnth | +6mnth |  |
| RBWH/1 | 20 | 20 | 20 | 20 | 20 | 20 | 20 | 140 |
| RBWH/2 | 20 | 20 | 20 | 20 | 20 | 20 | 20 | 140 |
| RBWH/3 | 20 | 20 | 20 | 20 | 20 | 20 | 20 | 140 |
| RBWH/4 | 20 | 20 | 20 | 20 | 20 | 20 | 20 | 140 |
| QCH/1 | 20 | 20 | 20 | 20 | 20 | 20 | 20 | 140 |
| QCH/2 | 20 | 20 | 20 | 20 | 20 | 20 | 20 | 140 |
| QCH/3 | 20 | 20 | 20 | 20 | 20 | 20 | 20 | 140 |
| QCH/4 | 20 | 20 | 20 | 20 | 20 | 20 | 20 | 140 |
| GCUH/1 | 20 | 20 | 20 | 20 | 20 | 20 | 20 | 140 |
| GCUH/2 | 20 | 20 | 20 | 20 | 20 | 20 | 20 | 140 |
| GCUH/3 | 20 | 20 | 20 | 20 | 20 | 20 | 20 | 140 |
| GCUH/4 | 20 | 20 | 20 | 20 | 20 | 20 | 20 | 140 |
| **Total** | 240 | 240 | 240 | 240 | 240 | 240 | 240 | 1680 |

**Supplementary file 2. DART cluster-randomisation schema.**
