# Supplementary material for: Improving difficult peripheral intravenous access requires thought, training and technology (DART3): a stepped-wedge, cluster randomised controlled trial protocol
Source: BMC Health Serv Res. 2023 Jun 7;23:587. doi: 10.1186/s12913-023-09499-0 (PMC10249237; doi:10.1186/s12913-023-09499-0)
Supplement: Supplementary file 3 — Supplementary Material 3 [file 12913_2023_9499_MOESM3_ESM.pdf]

DART3

Difficult Access Requires:
Thought, Training and Technology

STATISTICAL ANALYSIS PLAN

Version 1.0

27 February 2023

Author: Emily Young, reviewed by Prof Rob Ware

Study identifiers:

NHMRC ID: APP1180193
ANZCTR: ACTRN12621001497897

STUDY INVESTIGATORS

Prof Claire Rickard
Ms Christine Brown
Prof Nicole Marsh
Ms Tricia Kleidon
Prof Gerben Keijzers
Dr Jessica Schults
Dr Amanda Corley
Assoc Prof Lisa Hall
Assoc Prof Pauline Calleja
Dr Frances Williamson
Assoc Prof Steven McTaggart
Dr Alison Craswell
Prof Amanda Ullman
Ms Zhen Lin
Prof Joshua Byrnes
Prof Marie Cooke
Prof Robert Ware
Ms Emily Young
Dr Louise Cullen
Dr Nathan Peters
Dr Stuart Watkins
Dr Fiona Macfarlane
Mr Callan Battley

TABLE OF CONTENTS

List of abbreviations ... 4

1. Administrative information ... 5

  1.1 Study identifiers ... 5

  1.2 Contributors to the statistical analysis plan ... 5

2. Study synopsis ... 6

  2.1 Study objectives ... 6

    2.1.1 Primary hypothesis ... 6

    2.1.2 Secondary hypotheses ... 6

  2.2 Patient population ... 7

    2.2.1 Inclusion criteria ... 7

    2.2.2 Exclusion criteria ... 7

  2.3 Outcomes ... 7

    2.3.1 Primary outcome ... 7

    2.3.2 Secondary outcomes ... 8

  2.4 Intervention ... 9

  2.5 Randomisation and blinding ... 10

  2.6 Sample size ... 10

3 Statistical analysis ... 15

  3.1 General principles ... 15

    3.1.1 Statistical software ... 15

    3.1.2 Reporting conventions ... 15

    3.1.3 Data cleaning approach ... 15

    3.1.4 Data definitions/derivations including, but not limited to, visit windows definitions ... 15

    3.1.5 Confidence intervals and P-values ... 15

  3.2 Interim analyses ... 15

  3.3 Multiplicity adjustment ... 15

  3.4 Data sets to be analysed: Adherence and protocol deviations ... 16

  3.5 Subject disposition ... 16

  3.6 Patient characteristics and baseline comparisons ... 19

  3.7 Compliance to study intervention(s) ... 22

  3.8 Analysis of the primary outcome ... 23

    3.8.1 Primary outcome ... 23

    3.9.1 Secondary outcome 1 ... 23

    3.9.2 Secondary outcome 2 ... 24

    3.9.3 Secondary outcome 3 ... 24

3.9.4 Secondary outcome 4 25
3.9.5 Secondary outcome 5 26
3.9.6 Secondary outcome 6 26
3.9.7 Secondary outcome 7 27
3.9.8 Secondary outcome 8 27
3.9.9 Secondary outcome 9 28
3.9.10 Secondary outcome 10 28
3.9.11 Secondary outcome 11 29
3.9.12 Secondary outcome 12 30
3.9.13 Secondary outcome 13 30
3.9.14 Secondary outcome 14 31
3.9.15 Secondary outcome 15 31
3.9.16 Secondary outcome 16 32
3.9.17 Secondary outcome 17 33
4. References 33
4.1 Nonstandard statistical methods 33
4.2 Other standard operating procedures or documents to be adhered to: 33
Appendix 1: Proposed tables and figures 34

List of abbreviations

BSI	Blood stream infection
DART^3	Difficult Access Requires Thought, Training, and Technology
DIVA	Difficult intravenous access
IQR	Interquartile range
PIVC	Peripheral intravenous catheter
ReN	Research nurse
SD	Standard deviationr

1. Administrative information

1.1 Study identifiers

**Protocol:**	Version 1.0; Date: 19th May 2021
**ANZCTR ID:**	ACTRN12621001497897
**NHMRC ID:**	APP1180193

1.2 Contributors to the statistical analysis plan

The undersigned have reviewed this plan and approve it as final. They find it to be consistent with the requirements of the protocol as it applies to their respective areas. They also find it to be compliant with ICH-E9 principles and, in particular, confirm that this analysis plan was developed in a completely blinded manner (i.e., without knowledge of the effect of the intervention(s) being assessed).

**Name**	**Signature**	**Date**
Professor Claire Rickard<br>Principal Investigator		Date
Professor Robert Ware<br>Professor of Biostatistics		28/02/2023
Ms Christine Brown<br>Project Manager		03/03/2023
Ms Emily Young<br>Statistician		27/02/2023

2. Study synopsis

DART[3] is a multi-centre stepped wedge cluster randomised trial designed to determine the extent to which first attempt peripheral intravenous catheter (PIVC) insertion success, and other associated outcomes, are improved among in Difficult Intravenous Access (DIVA), and non-DIVA patients, within hospital wards and departments using the DIVA Identification and Ultrasound Escalation Pathways, compared with hospital wards and departments offering usual care.

2.1 Study objectives

(1) To develop hospital-based interventions that support DIVA identification and escalation, adapted to the local health care context.

(2) To implement co-designed DIVA identification and escalation pathways, and evaluate their clinical effectiveness, cost effectiveness and implementation strategies.

(3) Develop recommendations for future strategic options for scale-up and optimised implementation of DIVA identification and escalation pathways in different settings.

2.1.1 Primary hypothesis

The primary objective is to determine the extent to which implementation of the DIVA Identification and Ultrasound Escalation Pathways impacts first attempt PIVC insertion success for DIVA patients.

H_1: Implementation of the DIVA Identification and Ultrasound Escalation Pathways will significantly increase first attempt PIVC insertion success **for DIVA patients**

2.1.2 Secondary hypotheses

The secondary objectives are to determine the extent to which implementation of the DIVA Identification and Ultrasound Escalation Pathways: impacts first attempt PIVC insertion success for all patients; is feasible; is sustainable; is cost-effective; impacts incidence of ultrasound-guided insertion among DIVA patients; impacts number of PIVC insertion attempts; impacts rates of successful PIVC placement and time-to-therapy; impacts post-insertion failure and complications; and impacts patient/carer and staff satisfaction with insertion procedure.

Implementation of the DIVA Identification and Ultrasound Escalation Pathways:

$H_{2.1}$: Will significantly increase first attempt PIVC insertion success **for all patients**

$H_{2.2}$: Increases the proportion of DIVA patients with ultrasound used at first, or any attempt

$H_{2.3}$: Reduces the number of PIVC insertion attempts

$H_{2.4}$: Results in higher rates of successful PIVC placement

$H_{2.5}$: Results in shorter time-to-therapy

$H_{2.6}$: Results in higher rates of alternate device use

$H_{2.7}$: Results in higher rates of alternate route use (e.g., oral)

$H_{2.8}$: Reduces post-insertion PIVC failure

$H_{2.9}$: Reduces insertion/post-insertion complications

$H_{2.10}$: Results in increased PIVC dwell time

$H_{2.11}$: Is sustainable (i.e., there is no significant reduction in first time insertion success at +3 or +6 months compared to full implementation (month 10)

$H_{2.112}$: Reduces rates of unnecessary PIVCs

$H_{2.13}$: Is cost-effective

$H_{2.14}$: Reduces rates of cluster level primary blood stream infection (BSI) and S. Aureus BSI

$H_{2.15}$: Improves patient/carer satisfaction with insertion procedure

$H_{2.16}$: Reduces patient/carer pain with insertion procedure

$H_{2.17}$: Improves staff satisfaction with insertion procedure

2.2 Patient population

2.2.1 Inclusion criteria

Cluster eligibility is emergency department, inpatient ward, or day procedure unit where >10 PIVCs/week are typically inserted. Across each cluster at each participating hospital, any PIVC, inclusive of short and long PIVC or midline catheter, being inserted may be considered for inclusion. Patient eligibility: Patient (DIVA or non-DIVA) of any age (neonate to elderly) prescribed PIVC insertion.

2.2.2 Exclusion criteria

Exclusion criteria are emergencies (e.g., Medical Emergency Team call) requiring intraosseous access. Exclusion areas are operating theatres, radiology, rehabilitation, or psychiatric units because they either have all expert inserters so have less insertion failure, or rarely insert PIVCs. Screening data will not be collected for excluded patients.

2.3 Outcomes

2.3.1 Primary outcome

The primary outcome is first attempt PIVC insertion success for DIVA patients. The primary outcome is defined as one needle puncture, by one inserter, to achieve PIVC insertion for DIVA patients.

Data for the primary outcome will be collected in 2-month increments during the baseline and implementation phases of the study (corresponding to each study step), and 3-month increments

during the sustainability phase of the study. During each window of time (or step), data will be collected at randomly selected study wards on specific days throughout the time-period. All outcomes will be analysed collectively once data collection has been completed.

2.3.2 Secondary outcomes

Secondary outcome 1 is first attempt PIVC insertion success for all patients. Secondary outcome 1 is defined as one needle puncture, by one inserter, to achieve PIVC insertion for all patients.

Secondary outcome 2 is ultrasound adoption. Secondary outcome 2 is defined as the proportion of DIVA patients with ultrasound used at first, or any attempt.

Secondary outcome 3 is number of PIVC insertion attempts. Secondary outcome 3 is defined as numbers of skin punctures to attempt PIVC placement.

Secondary outcome 4 is successful procedural outcome defined as successful PIVC insertion [4]

Secondary outcome 5 is time from PIVC referral to PIVC insertion (censored at 48 hours).

Secondary outcome 6 is use of alternate device.

Secondary outcome 7 is use of alternate route (e.g., oral).

Secondary outcome 8 is PIVC failure. Secondary outcome 11 is defined as a composite measure of local infection, primary bloodstream infection, occlusion, infiltration/extravasation, dislodgement (includes leaking), thrombosis and/or phlebitis.

Secondary outcome 9 is insertion/post-insertion complications. Secondary outcome 9 is defined as bruising, haematoma, nerve injury, arterial puncture, or skin injury as well as the individual components of PIVC failure (above).

Secondary outcome 10 is PIVC dwell time. Secondary outcome 10 is defined as time from PIVC insertion to PIVC removal (in hours).

Secondary outcome 11 is sustainability of first attempt insertion success after implementation of the DIVA Identification and Escalation Pathways. Sustainability is defined as first attempt insertion success at +3 or +6 months compared to full implementation (month 10).

Secondary outcome 12 is PIVC necessity. Secondary outcome 12 is defined as PIVC used for fluids or medications within 24 hours (excluding patients who require prophylactic PIVC *in situ* as part of their treatment e.g., status epilepticus).

Secondary outcome 13 is cluster level routinely collected rates of primary BSI and S. Aureus BSI.

Secondary outcome 14 is patient/carer/parent satisfaction with insertion procedure. Secondary outcome 14 is defined according to a 0-10 numeric rating scale.

Secondary outcome 15 is patient/carer/parent pain with insertion procedure. Secondary outcome 15 is defined according to a 0-10 numeric rating scale.

Secondary outcome 16 is inserter (initial, successful, and/or abandoned inserter) satisfaction with insertion procedure. Secondary outcome 16 is defined according to a 0-10 numeric rating scale.

As with the primary outcome, data on secondary outcomes will be collected in 2-month increments during the baseline and implementation phases of the study (corresponding to each study step), and 3-month increments during the sustainability phase of the study. During each window of time (or step), data will be collected at randomly selected study wards on specific days throughout the time-period. All outcomes will be analysed collectively once data collection has been completed.

2.4 Intervention

A stepped-wedge cluster randomised trial will be used to implement and evaluate the DIVA Identification and Ultrasound Escalation Pathways at 3 South East Queensland hospitals. Each hospital will utilise 4 wards/departments for a total of 12 clusters. Within each cluster, 20 patients undergoing PIVC insertion procedures will be recruited. Sustainability will be measured at 3 and 6 months post full implementation. In total, it is expected that 600 patients will be recruited into the control condition, and 1080 patients will be recruited into the intervention condition.

In the control steps (Figure 1.), clusters will continue usual care. This will vary by site, however, in general usual care will be landmark insertion by default; with no/minimally implemented pathway to assess level of difficulty; and ad hoc referral to ultrasound skilled practitioners, typically after failed landmark attempts. During implementation, Research Nurses (ReNs) will work with investigators, local educators, and clinical managers to deliver implementation strategies to embed the DIVA Identification and Ultrasound Escalation Pathways (Figure 2.). All staff will be trained in the use of the DIVA tool and Escalation Pathway via a mix of written, didactic, and online learning modalities. ReNs will act as 'change champions', promoting consistent use of the tool and pathway. Advanced inserters (capable of ultrasound guided PIVC insertion) will be identified in the Escalation Pathway and be available to the study wards to provide a point of escalation for DIVA patients. Inserters will be the local accredited workforce (not ReNs).

Hospital/Dept.	Baseline	Implementation				Sustainability		Total
		Step 1	Step 2	Step 3	Step 4			
Month	1-2	3-4	5-6	7-8	9-10	+3mnth	+6mnth	
RBWH/1	20	20	20	20	20	20	20	140
RBWH/2	20	20	20	20	20	20	20	140
RBWH/3	20	20	20	20	20	20	20	140
RBWH/4	20	20	20	20	20	20	20	140
QCH/1	20	20	20	20	20	20	20	140
QCH/2	20	20	20	20	20	20	20	140
QCH/3	20	20	20	20	20	20	20	140
QCH/4	20	20	20	20	20	20	20	140
GCUH/1	20	20	20	20	20	20	20	140
GCUH/2	20	20	20	20	20	20	20	140
GCUH/3	20	20	20	20	20	20	20	140
GCUH/4	20	20	20	20	20	20	20	140
Total	240	240	240	240	240	240	240	1680

Figure 1. Stepped-wedge, cluster-randomisation scheme

2.5 Randomisation and blinding

As displayed in Figure 1., all 12 hospital clusters (wards/departments) will start the trial in the control phase for two months, with baseline measures taken. Following this, one cluster per hospital will randomly (computer generated) step-up to implementation each month over an 8-month period, until all are fully implemented. Sustainability will be measured by proportion of first-time insertion success and proportion following the pathway, at 3 and 6 months post full implementation. At each time point, a random sample of 20 PIVC insertion procedures will be studied per cluster. This will be achieved by randomly selecting dates for data collection, rostering ReNs on those dates and collecting the first 20 PIVC insertions that occur sequentially while the ReN is available. To fully understand health system implementation, we will assign up to one in four ReN shifts to occur in the early mornings, evenings or at weekends.

2.6 Sample size

The study has been designed to have adequate statistical power to test the primary hypothesis. We expect that approximately 40% of recruited patients will be DIVAs (8 per cluster sample) and 60% non-DIVAs (12 per cluster sample). Using Hemming's method for cluster randomised controlled trials, including correction for intra class correlation (r=0.03 constant throughout study), a two-sample test of proportions with 4 steps, 3 clusters randomised at each step, and 20 patients/cluster (Hemming & Girling, 2018). For patients classified as DIVA, we assume the current percentage of first attempt insertion success of 50% (based on prior meta-analysis (van Loon, 2018) and consistent with our pilot (Jauncey-Cooke, 2018), and that there will be a 50% relative improvement to 75% of first insertion success. With 20 patients recruited per cluster we will have >90% power to detect a statistically significant difference in first attempt insertion success before and after exposure to the intervention for DIVA patients (alpha=0.05).

When considering the effect of the intervention on all recruited patients, we assume the current first insertion success percentage in non-DIVA patients is 70%, and that this will rise to 80% post-intervention. This is equivalent to an overall change from 62% first attempt insertion success to 78% first attempt insertion success. With 240 patients at each of the 5 data collection periods (baseline plus 4 steps), we have >90% power to detect a statistically significant difference between pre- and post-implementation of pathways (alpha=0.05).

To measure sustainability of the primary endpoint we will continue to assess 240 patients at each sustainability time point (3 and 6 months). We will have data on 168 DIVA patients receiving the pathway at steps 3 and 4, and 192 DIVA patients at the 3- and 6-month follow-up periods combined. This will allow the identification of a drop in first attempt insertion success from 75% to <60% post-intervention with 80% power (alpha=0.05) (van Loon, 2018; Jauncey-Cooke 2018). When considering all patients regardless of DIVA status we have 420 at steps 3 and 4 and 480 at follow-up, giving 80% power to identify an overall reduction in first time insertion success from 78% to <70% post-intervention (alpha=0.05).

This leads to a total sample size of 1680 observations, consisting of 240 observations at each of the 7 periods (baseline, steps 1-4, 3- and 6-month follow-up).

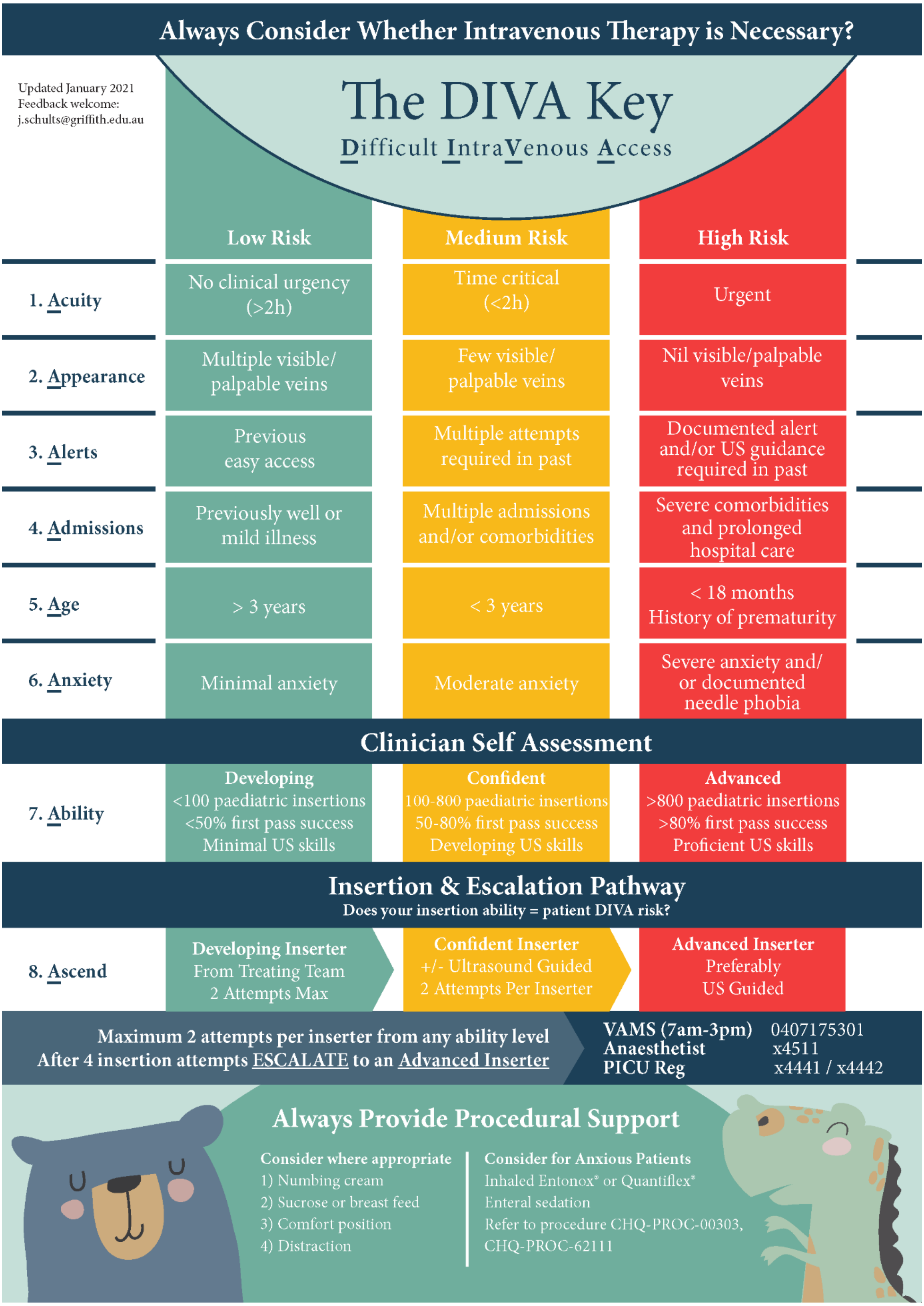

Figure 2. Queensland Children's Hospital DIVA Key & Escalation Pathway

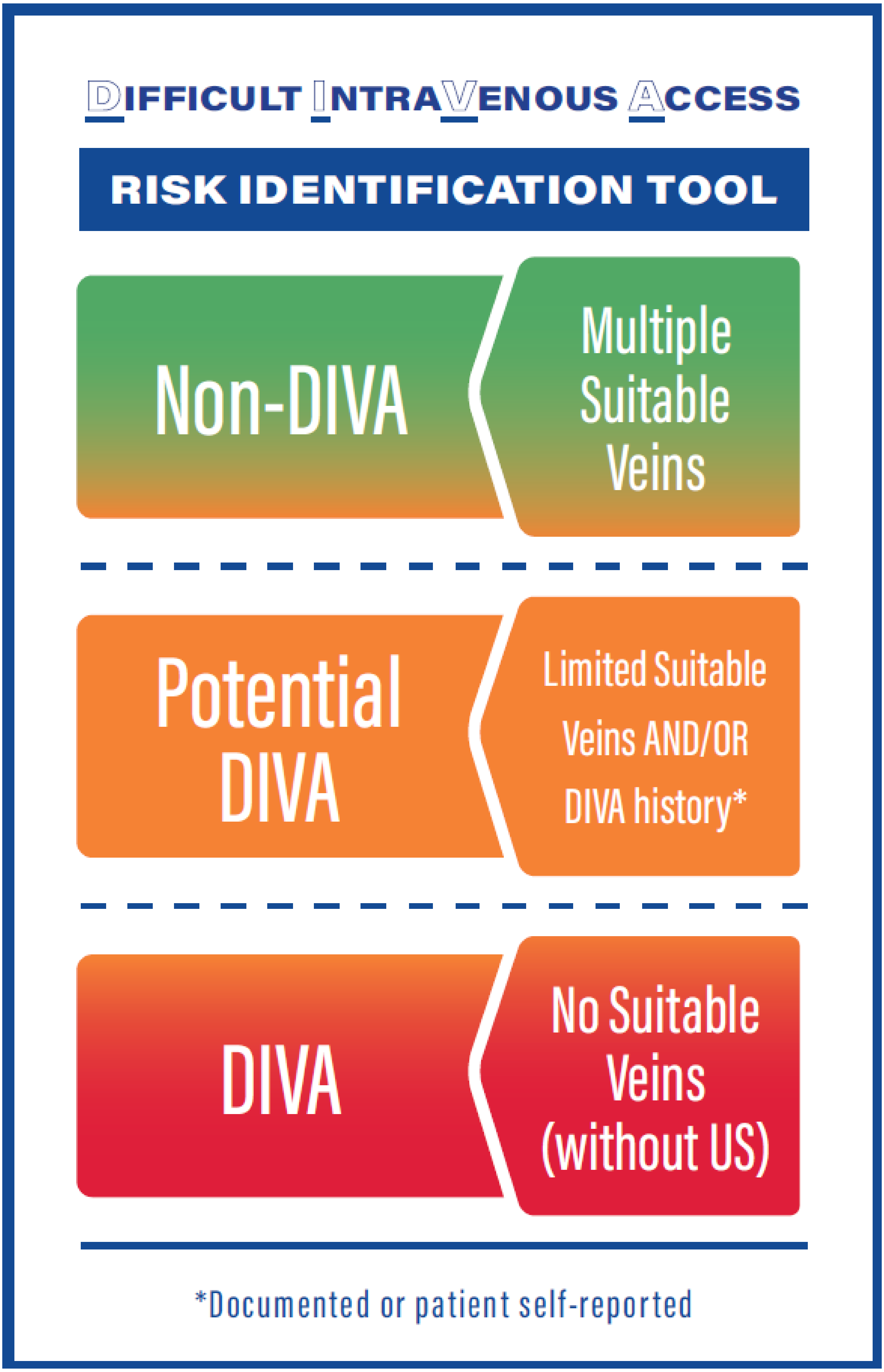

Figure 3. Royal Brisbane and Women's Hospital DIVA Key

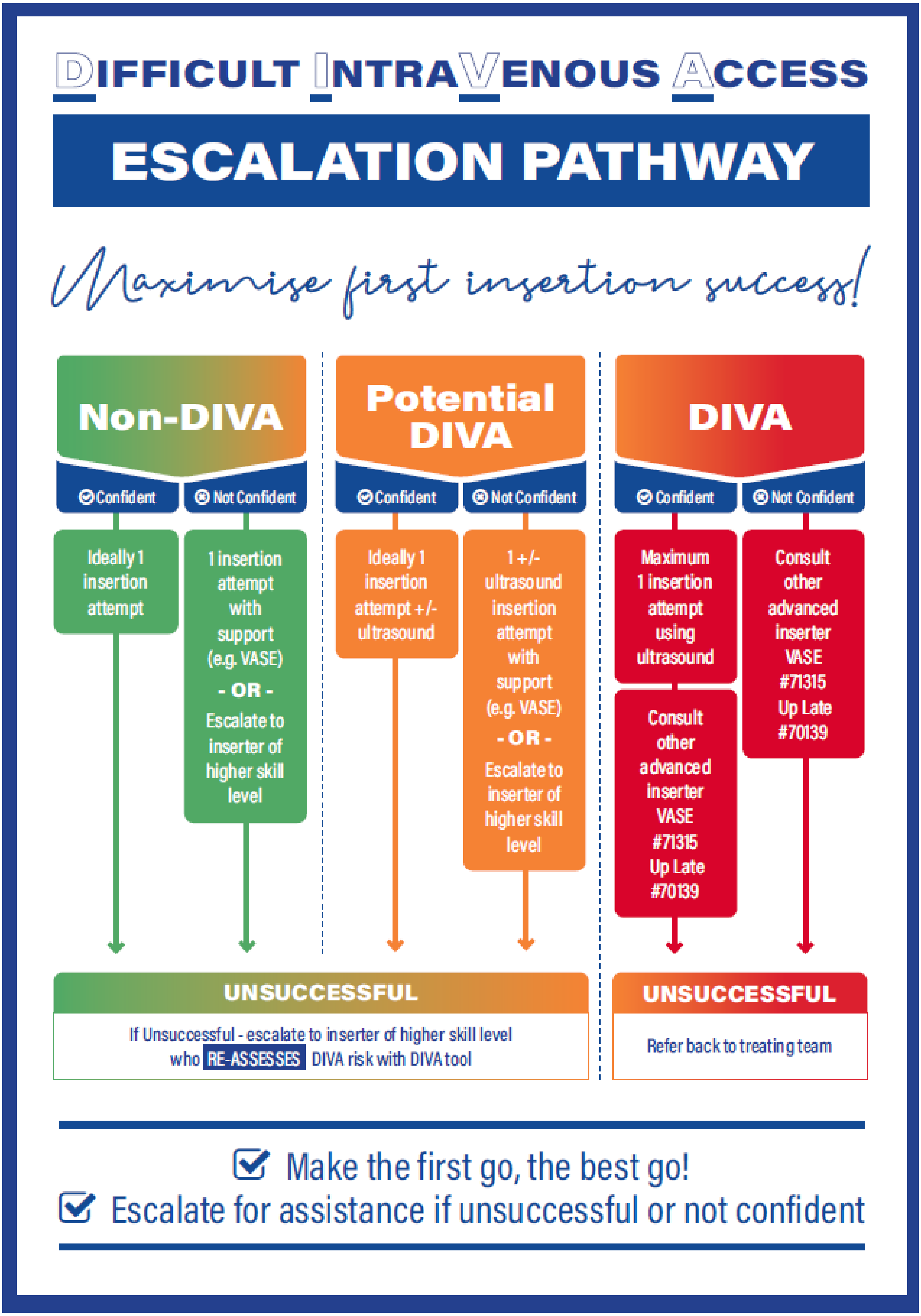

Figure 4. Royal Brisbane and Women's Hospital DIVA Escalation Pathway

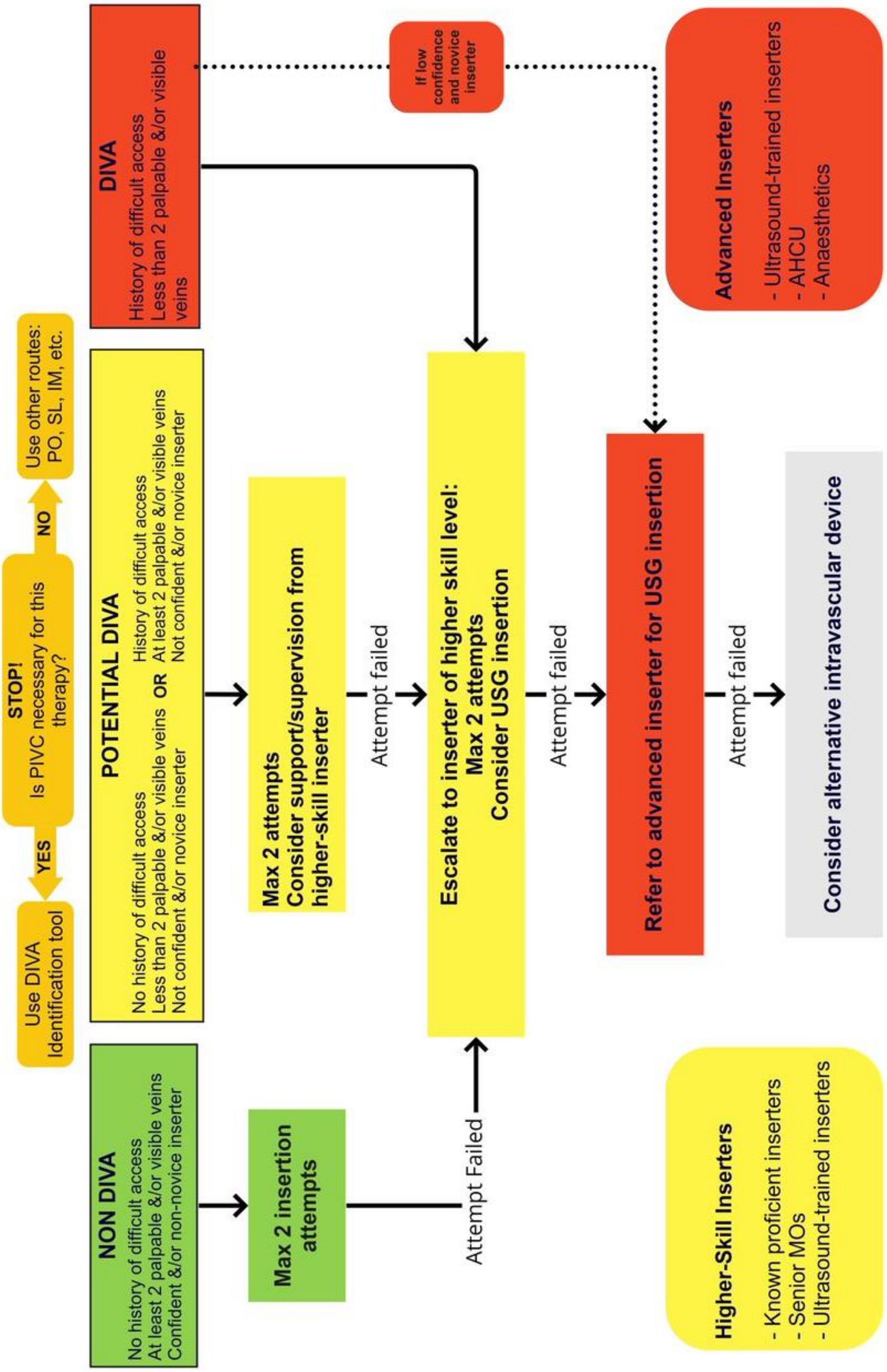

Figure 5. Gold Coast University Hospital DIVA Key & Escalation Pathway

3 Statistical analysis

3.1 General principles

3.1.1 Statistical software

All statistical analyses will be performed using Stata v13 (StataCorp. *Stata Statistical Software.* College Station, TX: StataCorp LP).

3.1.2 Reporting conventions

P-values will be reported to 2 decimal places to p=0.01, then 3 decimal places to p=0.001. All smaller values will be reported as p<0.001.

3.1.3 Data cleaning approach

Data collection and quality management will be completed through use of the REDCap database.

3.1.4 Data definitions/derivations including, but not limited to, visit windows definitions

Data collection will be classified by study steps, which will be defined according to calendar month.

3.1.5 Confidence intervals and P-values

For each outcome variable, statistical significance will be assessed at the 0.05 level and 95% confidence intervals will be reported.

3.2 Interim analyses

No interim analyses will be carried out for this study.

3.3 Multiplicity adjustment

No formal adjustments for multiplicity are planned, however significant test results will be interpreted considering the multiple comparisons made. To express uncertainty about the treatment effect, 95% confidence intervals will be used.

3.4 Data sets to be analysed: Adherence and protocol deviations

The primary analyses will be ‘intention-to-treat’.

Adherence to the intervention will be defined as the successful delivery of implementation strategies by ReNs, investigators, local educators, and clinical managers to embed the DIVA Identification and Ultrasound Escalation Pathways. Adherence to the intervention will be assessed by the extent to which application of the DIVA Escalation Pathway reflects the study protocol.

Protocol deviations will be defined as inclusion of ineligible patients, recruitment outside designated timeframe, monitoring is missed, or any other deviations from the study protocol.

All protocol deviations will be summarised by type, including a description of the deviation and the corrective action taken.

3.5 Subject disposition

Details on recruitment are outlined in the participant flowchart (Figure 6).

Consent will not be sought due to the observational and quality improvement nature of the study, and routine collection of these data in the provision of patient care. As such, participants will not be eligible to withdraw from the intervention. Loss to follow-up is considered unlikely and not expected to significantly impact data collection.

Should loss to follow-up occur prior to completion of the DIVA Identification and Escalation Pathways a new patient will be recruited in replacement ensuring potential for complete data collection for H_{1-8}. In the unlikely event that loss to follow-up should occur after completion of the Escalation Pathway but prior to device removal, data collection for H_{9-10} may be impacted.

Any events of loss to follow-up will be summarised by reason, alongside any resulting study amendments.

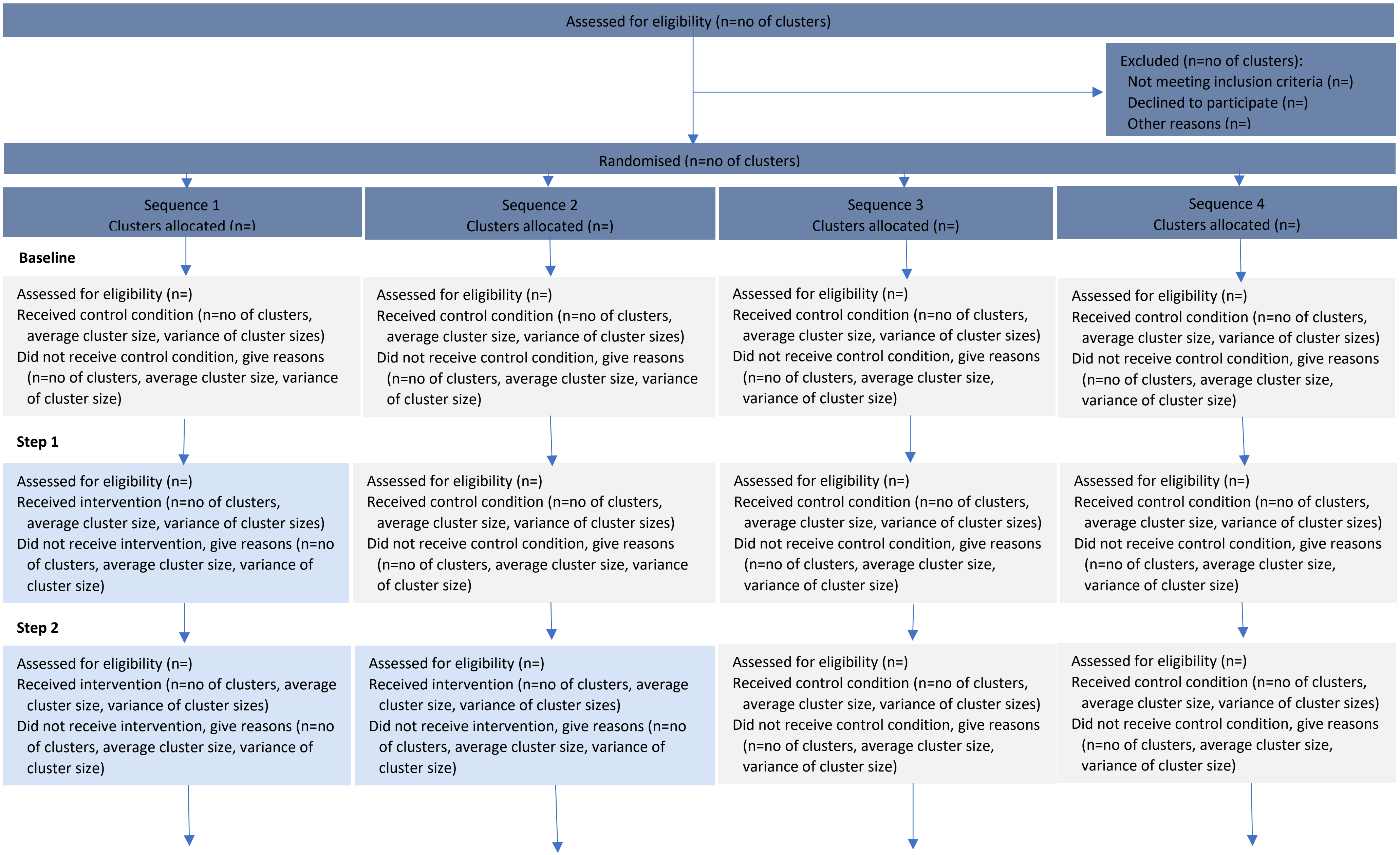
Assessed for eligibility (n=no of clusters)
Excluded (n=no of clusters): Not meeting inclusion criteria (n=) Declined to participate (n=) Other reasons (n=)
Randomised (n=no of clusters)
Sequence 1 Clusters allocated (n=)
Sequence 2 Clusters allocated (n=)
Sequence 3 Clusters allocated (n=)
Sequence 4 Clusters allocated (n=)
Baseline
Assessed for eligibility (n=) Received control condition (n=no of clusters, average cluster size, variance of cluster sizes) Did not receive control condition, give reasons (n=no of clusters, average cluster size, variance of cluster size)
Assessed for eligibility (n=) Received control condition (n=no of clusters, average cluster size, variance of cluster sizes) Did not receive control condition, give reasons (n=no of clusters, average cluster size, variance of cluster size)
Assessed for eligibility (n=) Received control condition (n=no of clusters, average cluster size, variance of cluster sizes) Did not receive control condition, give reasons (n=no of clusters, average cluster size, variance of cluster size)
Assessed for eligibility (n=) Received control condition (n=no of clusters, average cluster size, variance of cluster sizes) Did not receive control condition, give reasons (n=no of clusters, average cluster size, variance of cluster size)
Step 1
Assessed for eligibility (n=) Received intervention (n=no of clusters, average cluster size, variance of cluster sizes) Did not receive intervention, give reasons (n=no of clusters, average cluster size, variance of cluster size)
Assessed for eligibility (n=) Received control condition (n=no of clusters, average cluster size, variance of cluster sizes) Did not receive control condition, give reasons (n=no of clusters, average cluster size, variance of cluster size)
Assessed for eligibility (n=) Received control condition (n=no of clusters, average cluster size, variance of cluster sizes) Did not receive control condition, give reasons (n=no of clusters, average cluster size, variance of cluster size)
Assessed for eligibility (n=) Received control condition (n=no of clusters, average cluster size, variance of cluster sizes) Did not receive control condition, give reasons (n=no of clusters, average cluster size, variance of cluster size)
Step 2
Assessed for eligibility (n=) Received intervention (n=no of clusters, average cluster size, variance of cluster sizes) Did not receive intervention, give reasons (n=no of clusters, average cluster size, variance of cluster size)
Assessed for eligibility (n=) Received intervention (n=no of clusters, average cluster size, variance of cluster sizes) Did not receive intervention, give reasons (n=no of clusters, average cluster size, variance of cluster size)
Assessed for eligibility (n=) Received control condition (n=no of clusters, average cluster size, variance of cluster sizes) Did not receive control condition, give reasons (n=no of clusters, average cluster size, variance of cluster size)
Assessed for eligibility (n=) Received control condition (n=no of clusters, average cluster size, variance of cluster sizes) Did not receive control condition, give reasons (n=no of clusters, average cluster size, variance of cluster size)

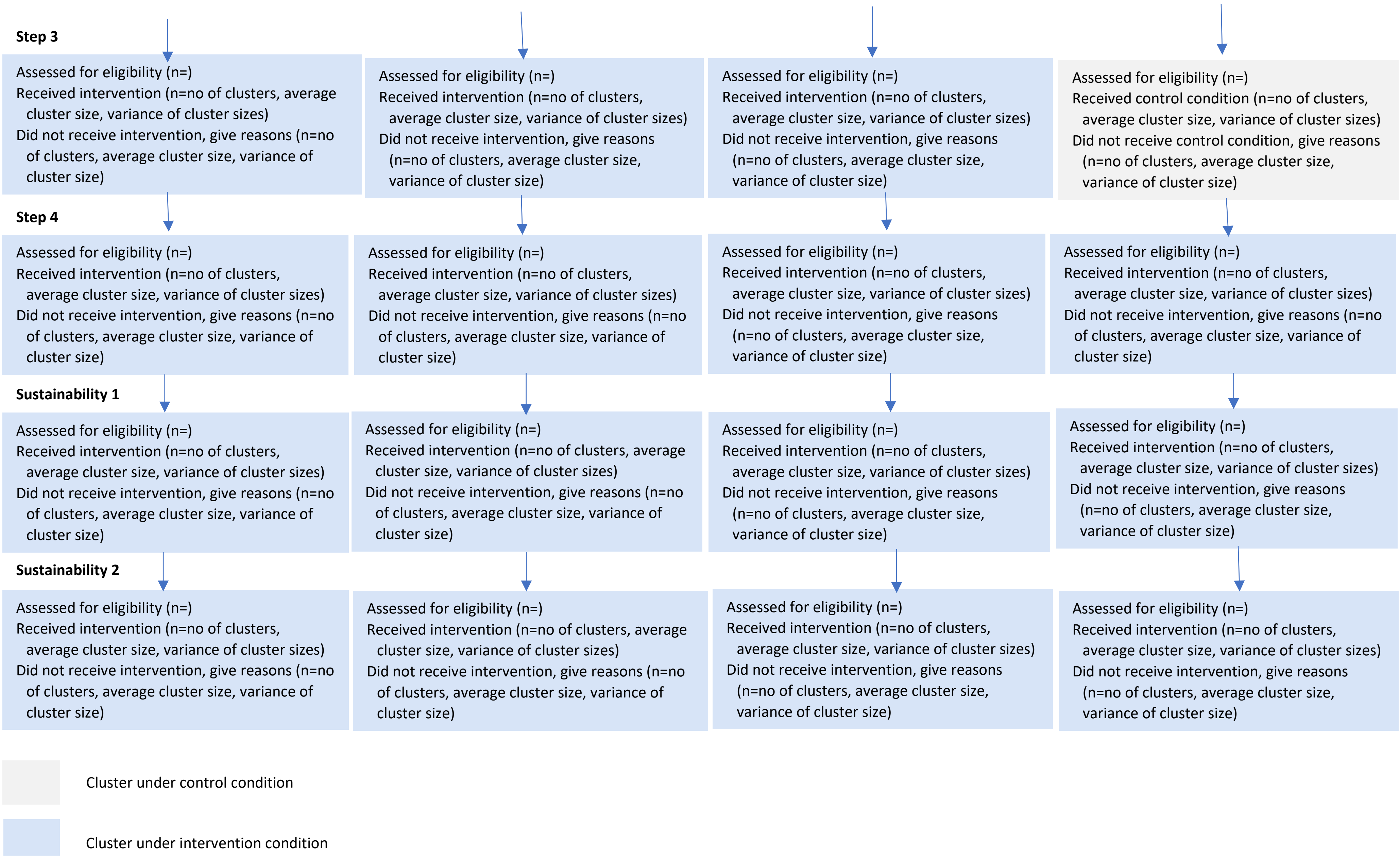

Figure 6: Participant flowchart

3.6 Patient characteristics and baseline comparisons

A descriptive comparison of the patients observed under each condition will be conducted on the baseline characteristics presented in the following table.

**Baseline characteristic**	**Categories**
Age - years	-
Number of PIVCs by Study site	-
Number of PIVCs by Cluster	-
Sex	Male
	Female
	Other
DIVA Tool Outcomes by Study Site and Cluster	-
Type of admission	Medical (Emergent)
	Medical (Elective)
	Surgical (Emergent)
	Surgical (Elective)
	Trauma
	Oncology/Haematology
	Other
Number of comorbidities:	0
	1
	2
	3
	>3
Bodyweight	Obese (BMI ≥30)
	Overweight (BMI ≥25 to <30)
	Normal weight (BMI ≥18.5 to <25)
	Underweight (BMI <18.5)

Infection at recruitment	Yes
	No
Pre-existing wound	Yes
	No
Skin integrity	Good
	Fair
	Poor
Skin type	Pale white
	White
	Light brown
	Moderate brown
	Dark brown
	Deeply pigmented dark
Intravenous antibiotics during PIVC dwell	Yes
	No
Previous PIVC inserted during admission	Yes
	No
Other Indwelling Vascular Devices	Central venous catheter/Peripherally inserted central-line catheter
	Midline
	Port
Other Indwelling Devices:	Wound Drain
	Stoma
	Tracheostomy
Reason for PIVC insertion:	Fluids
	Medication
	Blood Products
	Prophylactic Insertion
	Blood Sampling
	Other
Dominant side insertion	Yes

	No
	Ambidextrous
First-insertion Success (by phase, cluster, and site)	Yes
	No
PIVC inserted by	Vascular Access Specialist
	Nurse
	Medical officer
	Other
Insertion site	Anterior upper forearm
	Posterior lower forearm
	Wrist
	Posterior upper forearm
	Hand
	Cubital fossa
	Anterior lower forearm
	Anterior upper arm
	Posterior upper arm
Single insertion attempt	Yes
	No
Bruised insertion site	Yes
	No
Intravenous cannula size	22G
	20G
	18G
10-15 cm extension tubing	Yes
	No
3-way connector	Yes
	No
Successful PIVC Insertion (by phase, site, and cluster)	Yes
	No

Patient Satisfaction (frequency of each category by phase, site, and cluster)	-
Inserter Satisfaction (frequency of each category by phase, site, and cluster)	-
Ultrasound use for first insertion attempt (by phase, site, and cluster)	Yes<br>No
Complications (by phase, site, and cluster):	Arterial Puncture<br>Nerve Injury<br>Haematoma<br>Bruising<br>Prolonged Bleeding<br>Other
Attempt which complications occurred on:	First<br>Second<br>Third<br>Fourth<br>Fifth<br>Other

Summary statistics will be presented as frequency [percentage] for categorical variables, and chi-square tests will be used to determine between-group differences. Continuous variables will be summarised as mean [standard deviation (SD)] or median [interquartile range (IQR)] where appropriate, with t-tests (or Mann-Whitney U test) used to determine between-group differences.

3.7 Compliance to study intervention(s)

Compliance to the study invention(s) includes:

i) The extent to which the intervention is implemented as intended (implementation fidelity), over time and across different sites.
ii) How staff understand and respond to the intervention, over time and across different sites.
iii) Context over time and across different sites and factors (including managerial, economic, organisational, and work level) that affect implementation.

Variables which will be used to define compliance during implementation include:

**dd_1st_diva_imp_sus**	First inserter's assessment of patient's DIVA status (as measured by the DIVA identification tool

**dd_success_diva_imp_sus**	Successful inserter's assessment of patient's DIVA status (as measured by the DIVA identification tool)
**dd_aband_diva_imp_sus**	Final unsuccessful inserter's assessment of patient's DIVA status (as measured by the DIVA identification tool)

Documentation of 'no assessment by inserter' will represent lack of compliance with study interventions.

3.8 Analysis of the primary outcome

3.8.1 Primary outcome

The secondary outcome of interest is first attempt PIVC insertion success among DIVA patients. Patients identified as either 'DIVA' or 'potential DIVA' in the DIVA identification and Escalation Pathways will be included in the subgroup analysis. The measurement to be used to summarise data is frequency [percentage]. To derive the outcome, calculations will be used to determine the cumulative incidence in each of the control and intervention conditions, as well as the difference between control and intervention conditions. The endpoint for the primary outcome will be collection of the last participant at sustainability timepoint 2.

3.8.1.1 Main analysis

The individual will be the unit of analysis. Between group comparison of first attempt PIVC insertion success among DIVA patients will use mixed-effects logistic regression with cluster included as a random effect to account for probable non-independence of observations within clusters. Fixed effects included in the model will be study phase and time. The primary analysis will be 'intention-to-treat'. The trial hypotheses will be evaluated through a framework of superiority. The effect estimate presented will be odds ratio with 95% confidence interval.

Independence will be explored through plotting residuals against predicted values and use of Breusch-Pagan tests. Multicollinearity will be explored through Stata's 'collin' command. Linearity between the log odds of the dependent variable and the independent variables will be tested through the Box-Tidwell test. If assumptions for the preferred analysis are not met nonparametric binary logistic regression model using the local likelihood logit estimation method will be used.

Missing data will be reported when evident. <5% missing data is expected for the primary outcome as additional observations will be recruited when an observation is found to have missing data related to the primary outcome.

3.9 Analysis of secondary outcomes

3.9.1 Secondary outcome 1

The secondary outcome of interest is first attempt PIVC insertion success among all patients. The measurement to be used is cumulative incidence [percentage]. To derive the outcome, calculations will be used to determine the cumulative incidence in each of the control and intervention conditions, as well as the difference between control and intervention conditions. The endpoint for secondary outcome 1 will be collection of the last participant at sustainability timepoint 2.

3.9.1.1 Main analysis

The individual will be the unit of analysis. Between group comparison of first attempt PIVC insertion success among all patients will use mixed-effects logistic regression with cluster included as a random effect to account for probable non-independence of observations within clusters. Fixed effects included in the model will be study phase and time. The primary analysis will be ‘intention-to-treat’. The trial hypotheses will be evaluated through a framework of superiority. The effect estimate presented will be odds ratio with 95% confidence interval.

Independence will be explored through plotting residuals against predicted values and use of Breusch-Pagan tests. Multicollinearity will be explored through Stata’s ‘collin’ command. Linearity between the log odds of the dependent variable and the independent variables will be tested through the Box-Tidwell test. If assumptions for the preferred analysis are not met, nonparametric binary logistic regression model using the local likelihood logit estimation method will be used

Missing data will be reported when evident. <10% is expected for secondary outcome 1. If missing data is ≥20% imputation will be used.

3.9.2 Secondary outcome 2

The outcome of interest is proportion of PIVC insertions having completed the DIVA Identification and Ultrasound Escalation Pathways. The measurement to be used is cumulative incidence [percentage]. To derive the outcome, calculations will be used to determine the cumulative incidence in the intervention condition. The endpoint for secondary outcome 2 will be collection of the last participant at sustainability timepoint 2.

3.9.2.1 Main analysis

The individual will be the unit of analysis. Proportion of PIVC insertions having completed the DIVA Identification and Ultrasound Escalation Pathways will be compared to the 80% feasibility threshold. The analysis will be ‘per protocol’. The trial hypotheses will be evaluated through a framework of non-inferiority.

Missing data will be reported when evident. <10% is expected for secondary outcome 2. If missing data is ≥20% imputation will be used.

3.9.3 Secondary outcome 3

The secondary outcome of interest is intervention sustainability at 3 and 6 months. The measurement to be used is cumulative incidence [percentage]. To derive the outcome, calculations will be used to determine the cumulative incidence in each of the control and intervention conditions, as well as the difference between control and intervention conditions. The endpoint for secondary outcome 3 will be collection of the last participant at sustainability timepoint 2.

3.9.3.1 Main analysis

The individual will be the unit of analysis. Follow-up to sustainability comparison of first attempt PIVC insertion success among DIVA patients and all patients will use mixed-effects logistic regression with cluster included as a random effect to account for probable non-independence of observations within clusters. Fixed effects included in the model will be study phase and time. The primary analysis will be 'intention-to-treat'. The trial hypotheses will be evaluated through a framework of non-inferiority. The effect estimate presented will be odds ratio with 95% confidence interval.

Independence will be explored through plotting residuals against predicted values and use of Breusch-Pagan tests. Multicollinearity will be explored through Stata's 'collin' command. Linearity between the log odds of the dependent variable and the independent variables will be tested through the Box-Tidwell test. If assumptions for the preferred analysis are not met, nonparametric binary logistic regression model using the local likelihood logit estimation method will be used

Missing data will be reported when evident. <10% is expected for secondary outcome 3. If missing data is ≥20% imputation will be used.

3.9.4 Secondary outcome 4

The secondary outcome of interest is proportion of DIVA patients with ultrasound used at first or any attempt. The measurement to be used is cumulative incidence [percentage]. To derive the outcome, calculations will be used to determine the cumulative incidence in each of the control and intervention conditions, as well as the difference between control and intervention conditions. The endpoint for secondary outcome 4 will be collection of the last participant at sustainability timepoint 2.

3.9.4.1 Main analysis

The individual will be the unit of analysis. Between group comparison of ultrasound use at first, or any attempt among DIVA patients will use mixed-effects logistic regression with cluster included as a random effect to account for probable non-independence of observations within clusters. Fixed effects included in the model will be study phase and time. The primary analysis will be 'intention-to-treat'. The trial hypotheses will be evaluated through a framework of superiority. The effect estimate presented will be odds ratio with 95% confidence interval.

Independence will be explored through plotting residuals against predicted values and use of Breusch-Pagan tests. Multicollinearity will be explored through Stata's 'collin' command. Linearity between the log odds of the dependent variable and the independent variables will be tested through the Box-Tidwell test. If assumptions for the preferred analysis are not met, nonparametric binary logistic regression model using the local likelihood logit estimation method will be used.

Missing data will be reported when evident. <10% is expected for secondary outcome 4. If missing data is ≥20% imputation will be used.

3.9.5 Secondary outcome 5

The secondary outcome of interest is number of PIVC insertion attempts. The measurement to be used is median count [IQR]. To derive the outcome, calculations will be used to determine the difference between control and intervention conditions. The endpoint for secondary outcome 5 will be collection of the last participant at sustainability timepoint 2.

3.9.5.1 Main analysis

The individual will be the unit of analysis. Between group comparison of number of PIVC insertion attempts will use mixed-effects linear regression with cluster included as a random effect to account for probable non-independence of observations within clusters. Fixed effects included in the model will be study phase (control/implementation) and time. The primary analysis will be 'intention-to-treat'. The trial hypotheses will be evaluated through a framework of superiority. The effect estimate presented will be mean difference with 95% confidence interval.

Linear relationships will be explored through scatter plots and Q-Q-Plots. Normality will be explored through histograms and Shapiro-Wilk tests. Homoscedasticity will be explored through a scatterplot of residuals versus predicted values. Independence will be explored through plotting residuals against predicted values and use of Breusch-Pagan tests. Errors in variables will be explored through Delgado and Gonzalez Manteiga's 'dgmtest' test in Stata. Multicollinearity will be explored through Stata's 'collin' command. If assumptions for the preferred analysis are not met, median regression will be substituted.

Missing data will be reported when evident. <10% is expected for secondary outcome 5. If missing data is ≥20% imputation will be used.

3.9.6 Secondary outcome 6

The secondary outcome of interest is rate of successful PIVC placement. The measurement to be used is cumulative incidence [percentage]. To derive the outcome, calculations will be used to determine the cumulative incidence in each of the control and intervention conditions, as well as the difference between control and intervention conditions. The endpoint for secondary outcome 6 will be collection of the last participant at sustainability timepoint 2.

3.9.6.1 Main analysis

The individual will be the unit of analysis. Between group comparison of successful PIVC placement will use mixed-effects logistic regression with cluster included as a random effect to account for probable non-independence of observations within clusters. Fixed effects included in the model will be study phase and time. The primary analysis will be 'intention-to-treat'. The trial hypotheses will be evaluated through a framework of superiority. The effect estimate presented will be odds ratio with 95% confidence interval.

Independence will be explored through plotting residuals against predicted values and use of Breusch-Pagan tests. Multicollinearity will be explored through Stata's 'collin' command. Linearity between the log odds of the dependent variable and the independent variables will be tested through the Box-Tidwell test. If assumptions for the preferred analysis are not met, nonparametric binary logistic regression model using the local likelihood logit estimation method will be used.

Missing data will be reported when evident <10% is expected for secondary outcome 6. If missing data is ≥20% imputation will be used.

3.9.7 Secondary outcome 7

The secondary outcome of interest is time-to-therapy. The measurement to be used is median elapsed time (in hours) [IQR]. To derive the outcome, calculations will be used to determine the time to event in each of the control and intervention conditions, as well as the difference between control and intervention conditions. The endpoint for secondary outcome 7 will be collection of the last participant at sustainability timepoint 2.

3.9.7.1 Main analysis

The individual will be the unit of analysis. Between group comparison of time-to-therapy will use a multilevel survival model with cluster included as a random effect to account for probable non-independence of observations within clusters. Fixed effects included in the model will be study phase and time. The primary analysis will be 'intention-to-treat'. The trial hypotheses will be evaluated through a framework of superiority. The effect estimate will be hazard ratio with 95% confidence interval.

Proportional hazards assumptions will be tested through Schoenfeld residuals. Nonlinearity will be assessed through martingale residuals. Influential observations will be examined through deviance residuals (or symmetric transformation of the martingale residuals). If assumptions for the preferred analysis are not met, Kaplan-Meier estimates and log-rank tests will be used.

Missing data will be reported when evident. <10% is expected for secondary outcome 7. If missing data is ≥20% imputation will be used.

3.9.8 Secondary outcome 8

The secondary outcome of interest is rate of alternate device use. The measurement to be used is cumulative incidence [percentage]. To derive the outcome, calculations will be used to determine the cumulative incidence in each of the control and intervention conditions, as well as the difference between control and intervention conditions. The endpoint for secondary outcome 8 will be collection of the last participant at sustainability timepoint 2.

3.9.8.1 Main analysis

The individual will be the unit of analysis. Between group comparison of alternate device use will use mixed-effects logistic regression with cluster included as a random effect to account for probable non-independence of observations within clusters. Fixed effects included in the model will be study phase and time. The primary analysis will be ‘intention-to-treat’. The trial hypotheses will be evaluated through a framework of superiority. The effect estimate presented will be odds ratio with 95% confidence interval.

Independence will be explored through plotting residuals against predicted values and use of Breusch-Pagan tests. Multicollinearity will be explored through Stata’s ‘collin’ command. Linearity between the log odds of the dependent variable and the independent variables will be tested through the Box-Tidwell test. If assumptions for the preferred analysis are not met, nonparametric binary logistic regression model using the local likelihood logit estimation method will be used.

Missing data will be reported when evident <10% is expected for secondary outcome 8. If missing data is ≥20% imputation will be used.

3.9.9 Secondary outcome 9

The secondary outcome of interest is rate of alternate route use. The measurement to be used is cumulative incidence [percentage]. To derive the outcome, calculations will be used to determine the cumulative incidence in each of the control and intervention conditions, as well as the difference between control and intervention conditions. The endpoint for secondary outcome 9 will be collection of the last participant at sustainability timepoint 2.

3.9.9.1 Main analysis

The individual will be the unit of analysis. Between group comparison of alternate route use will use mixed-effects logistic regression with cluster included as a random effect to account for probable non-independence of observations within clusters. Fixed effects included in the model will be study phase and time. The primary analysis will be ‘intention-to-treat’. The trial hypotheses will be evaluated through a framework of superiority. The effect estimate presented will be odds ratio with 95% confidence interval.

Independence will be explored through plotting residuals against predicted values and use of Breusch-Pagan tests. Multicollinearity will be explored through Stata’s ‘collin’ command. Linearity between the log odds of the dependent variable and the independent variables will be tested through the Box-Tidwell test. If assumptions for the preferred analysis are not met, nonparametric binary logistic regression model using the local likelihood logit estimation method will be used.

Missing data will be reported when evident <10% is expected for secondary outcome 9. If missing data is ≥20% imputation will be used.

3.9.10 Secondary outcome 10

The secondary outcome of interest is rate of unnecessary PIVCs. The measurement to be used is cumulative incidence [percentage]. To derive the outcome, calculations will be used to determine the cumulative incidence in each of the control and intervention conditions, as well as the difference between control and intervention conditions. The endpoint for secondary outcome 10 will be collection of the last participant at sustainability timepoint 2.

3.9.10.1 Main analysis

The individual will be the unit of analysis. Between group comparison of rates of unnecessary PIVC will use mixed-effects logistic regression with cluster included as a random effect to account for probable non-independence of observations within clusters. Fixed effects included in the model will be study phase and time. The primary analysis will be 'intention-to-treat'. The trial hypotheses will be evaluated through a framework of superiority. The effect estimate presented will be odds ratio with 95% confidence interval.

Independence will be explored through plotting residuals against predicted values and use of Breusch-Pagan tests. Multicollinearity will be explored through Stata's 'collin' command. Linearity between the log odds of the dependent variable and the independent variables will be tested through the Box-Tidwell test. If assumptions for the preferred analysis are not met, nonparametric binary logistic regression model using the local likelihood logit estimation method will be used.

Missing data will be reported when evident <10% is expected for secondary outcome 10. If missing data is ≥20% imputation will be used.

3.9.11 Secondary outcome 11

The secondary outcome of interest is post-insertion PIVC failure. The measurement to be used is elapsed time (in hours) [IQR]. To derive the outcome, calculations will be used to determine the time to event in each of the control and intervention conditions, as well as the difference between control and intervention conditions. The endpoint for secondary outcome 11 will be collection of the last participant at sustainability timepoint 2.

3.9.11.1 Main analysis

The individual will be the unit of analysis. Between group comparison of time-to-failure will use a multilevel survival model with cluster included as a random effect to account for probable non-independence of observations within clusters. Fixed effects included in the model will be study phase and time. The primary analysis will be 'intention-to-treat'. The trial hypotheses will be evaluated through a framework of superiority. The effect estimate will be hazard ratio with 95% confidence interval.

Proportional hazards assumptions will be tested through Schoenfeld residuals. Nonlinearity will be assessed through martingale residuals. Influential observations will be examined through deviance residuals (or symmetric transformation of the martingale residuals). If assumptions for the preferred analysis are not met, Kaplan-Meier estimates and log-rank tests will be used.

Missing data will be reported when evident. <10% is expected for secondary outcome 11. If missing data is ≥20% imputation will be used.

3.9.12 Secondary outcome 12

The secondary outcome of interest is PIVC dwell time. The measurement to be used is elapsed time (in hours) [IQR]. To derive the outcome, calculations will be used to determine the time to event in each of the control and intervention conditions, as well as the difference between control and intervention conditions. The endpoint for secondary outcome 12 will be collection of the last participant at sustainability timepoint 2.

3.9.12.1 Main analysis

The individual will be the unit of analysis. Between group comparison of dwell time will use a multilevel survival model with cluster included as a random effect to account for probable non-independence of observations within clusters. Fixed effects included in the model will be study phase and time. The primary analysis will be 'intention-to-treat'. The trial hypotheses will be evaluated through a framework of superiority. The effect estimate will be hazard ratio with 95% confidence interval.

Proportional hazards assumptions will be tested through Schoenfeld residuals. Nonlinearity will be assessed through martingale residuals. Influential observations will be examined through deviance residuals (or symmetric transformation of the martingale residuals). If assumptions for the preferred analysis are not met, Kaplan-Meier estimates and log-rank tests will be used.

Missing data will be reported when evident. <10% is expected for secondary outcome 12. If missing data is ≥20% imputation will be used.

3.9.13 Secondary outcome 13

The secondary outcome of interest is rate of insertion/post-insertion complications. The measurement to be used is cumulative incidence [percentage]. To derive the outcome, calculations will be used to determine the cumulative incidence in each of the control and intervention conditions, as well as the difference between control and intervention conditions. The endpoint for secondary outcome 13 will be collection of the last participant at sustainability timepoint 2.

3.9.13.1 Main analysis

The individual will be the unit of analysis. Between group comparison of insertion/post-insertion complications will use mixed-effects logistic regression with cluster included as a random effect to account for probable non-independence of observations within clusters. Fixed effects included in the model will be study phase and time. The primary analysis will be 'intention-to-treat'. The trial hypotheses will be evaluated through a framework of superiority. The effect estimate presented will be odds ratio with 95% confidence interval.

Independence will be explored through plotting residuals against predicted values and use of Breusch-Pagan tests. Multicollinearity will be explored through Stata's 'collin' command. Linearity between the log odds of the dependent variable and the independent variables will be tested through the Box-Tidwell test. If assumptions for the preferred analysis are not met, nonparametric binary logistic regression model using the local likelihood logit estimation method will be used.

Missing data will be reported when evident <10% is expected for secondary outcome 13. If missing data is ≥20% imputation will be used.

3.9.14 Secondary outcome 14

The secondary outcome of interest is patient/carer satisfaction with insertion procedure. The measurement to be used is a 0-10 rating on an 11-point Likert scale [IQR]. To derive the outcome, calculations will be used to determine the difference between control and intervention conditions. The endpoint for secondary outcome 14 will be collection of the last participant at sustainability timepoint 2.

3.9.14.1 Main analysis

The individual will be the unit of analysis. Between group comparison of patient/carer satisfaction with insertion procedure will use mixed-effects linear regression with cluster included as a random effect to account for probable non-independence of observations within clusters. Fixed effects included in the model will be study phase and time. The primary analysis will be 'intention-to-treat'. The trial hypotheses will be evaluated through a framework of superiority. The effect estimate will be mean (or median) difference with 95% confidence interval.

Linear relationships will be explored through scatter plots and Q-Q-Plots. Normality will be explored through histograms and Shapiro-Wilk tests. Homoscedasticity will be explored through a scatterplot of residuals versus predicted values. Independence will be explored through plotting residuals against predicted values and use of Breusch-Pagan tests. Errors in variables will be explored through Delgado and Gonzalez Manteiga's 'dgmtest' test in Stata. Multicollinearity will be explored through Stata's 'collin' command. If assumptions for the preferred analysis are not met, median regression will be substituted.

Missing data will be reported when evident. <10% is expected for secondary outcome 14. If missing data is ≥20% imputation will be used.

3.9.15 Secondary outcome 15

The secondary outcome of interest is patient/carer pain with insertion procedure. The measurement to be used is a 0-10 rating on an 11-point Likert scale [IQR]. To derive the outcome, calculations will be used to determine the difference between control and intervention conditions. The endpoint for secondary outcome 15 will be collection of the last participant at sustainability timepoint 2.

3.9.15.1 Main analysis

The individual will be the unit of analysis. Between group comparison of patient/carer pain with insertion procedure will use mixed-effects linear regression with cluster included as a random effect to account for probable non-independence of observations within clusters. Fixed effects included in the model will be study phase and time. The primary analysis will be ‘intention-to-treat’. The trial hypotheses will be evaluated through a framework of superiority. The effect estimate will mean (or median) difference with 95% confidence interval.

Linear relationships will be explored through scatter plots and Q-Q-Plots. Normality will be explored through histograms and Shapiro-Wilk tests. Homoscedasticity will be explored through a scatterplot of residuals versus predicted values. Independence will be explored through plotting residuals against predicted values and use of Breusch-Pagan tests. Errors in variables will be explored through Delgado and Gonzalez Manteiga’s ‘dgmtest’ test in Stata. Multicollinearity will be explored through Stata’s ‘collin’ command. If assumptions for the preferred analysis are not met, median regression will be substituted.

Missing data will be reported when evident. <10% is expected for secondary outcome 15. If missing data is ≥20% imputation will be used.

3.9.16 Secondary outcome 16

The secondary outcome of interest is staff satisfaction with insertion procedure. The measurement to be used is a 0-10 rating on an 11-point Likert scale [IQR]. To derive the outcome, calculations will be used to determine the difference between control and intervention conditions. The endpoint for secondary outcome 16 will be collection of the last participant at sustainability timepoint 2.

3.9.16.1 Main analysis

The individual will be the unit of analysis. Between group comparison of staff satisfaction with insertion procedure will use mixed-effects linear regression with cluster included as a random effect to account for probable non-independence of observations within clusters. Fixed effects included in the model will be study phase and time. The primary analysis will be ‘intention-to-treat’. The trial hypotheses will be evaluated through a framework of superiority. The effect estimate will mean (or median) difference with 95% confidence interval.

Linear relationships will be explored through scatter plots and Q-Q-Plots. Normality will be explored through histograms and Shapiro-Wilk tests. Homoscedasticity will be explored through a scatterplot of residuals versus predicted values. Independence will be explored through plotting residuals against predicted values and use of Breusch-Pagan tests. Errors in variables will be explored through Delgado and Gonzalez Manteiga’s ‘dgmtest’ test in Stata. Multicollinearity will be explored through Stata’s ‘collin’ command. If assumptions for the preferred analysis are not met, median regression will be substituted.

Missing data will be reported when evident. <10% is expected for secondary outcome 16. If missing data is ≥20% imputation will be used.

3.9.17 Secondary outcome 17

The secondary outcome of interest is rate of primary BSI and *S. Aureus* BSI. The measurement to be used is cumulative incidence [percentage]. To derive the outcome, calculations will be used to determine the cumulative incidence in each of the control and intervention conditions, as well as the difference between control and intervention conditions. The endpoint for secondary outcome 17 will be collection of the last participant at sustainability timepoint 2.

3.9.17.1 Main analysis

The individual will be the unit of analysis. Between group comparison of primary BSI and S. Aureus BSI will use mixed-effects logistic regression with cluster included as a random effect to account for probable non-independence of observations within clusters. Fixed effects included in the model will be study phase and time. The primary analysis will be 'intention-to-treat'. The trial hypotheses will be evaluated through a framework of superiority. The effect estimate presented will be odds ratio with 95% confidence interval.

Independence will be explored through plotting residuals against predicted values and use of Breusch-Pagan tests. Multicollinearity will be explored through Stata's 'collin' command. Linearity between the log odds of the dependent variable and the independent variables will be tested through the Box-Tidwell test. If assumptions for the preferred analysis are not met, nonparametric binary logistic regression model using the local likelihood logit estimation method will be used.

Missing data will be reported when evident <10% is expected for secondary outcome 17. If missing data is ≥20% imputation will be used.

4. References

4.1 Nonstandard statistical methods

Hemming K, Girling A. A Menu-Driven Facility for Power and Detectable-Difference Calculations in Stepped-Wedge Cluster-Randomized Trials. The Stata Journal: Promoting communications on statistics and Stata 2018; 14(2): 363-80.

4.2 Other standard operating procedures or documents to be adhered to:

Jauncey-Cooke J, et al. INTRO: insertion technology improves outcomes: ultrasound PIVC cannulation by nursing staff in Medical Day Units. ACCYPN. Perth; 2018

van Loon FHJ, Buise MP, Claassen JJF, Dierick-van Daele ATM, Bouwman ARA. Comparison of ultrasound guidance with palpation and direct visualisation for peripheral vein cannulation in adult patients: a systematic review and meta-analysis. Br J Anaesth 2018; 121(2): 358-66.

Appendix 1: Proposed tables and figures

Figure 1: Participant flowchart

Table 1: Baseline characteristics

Table 2: Analysis of primary and secondary outcomes

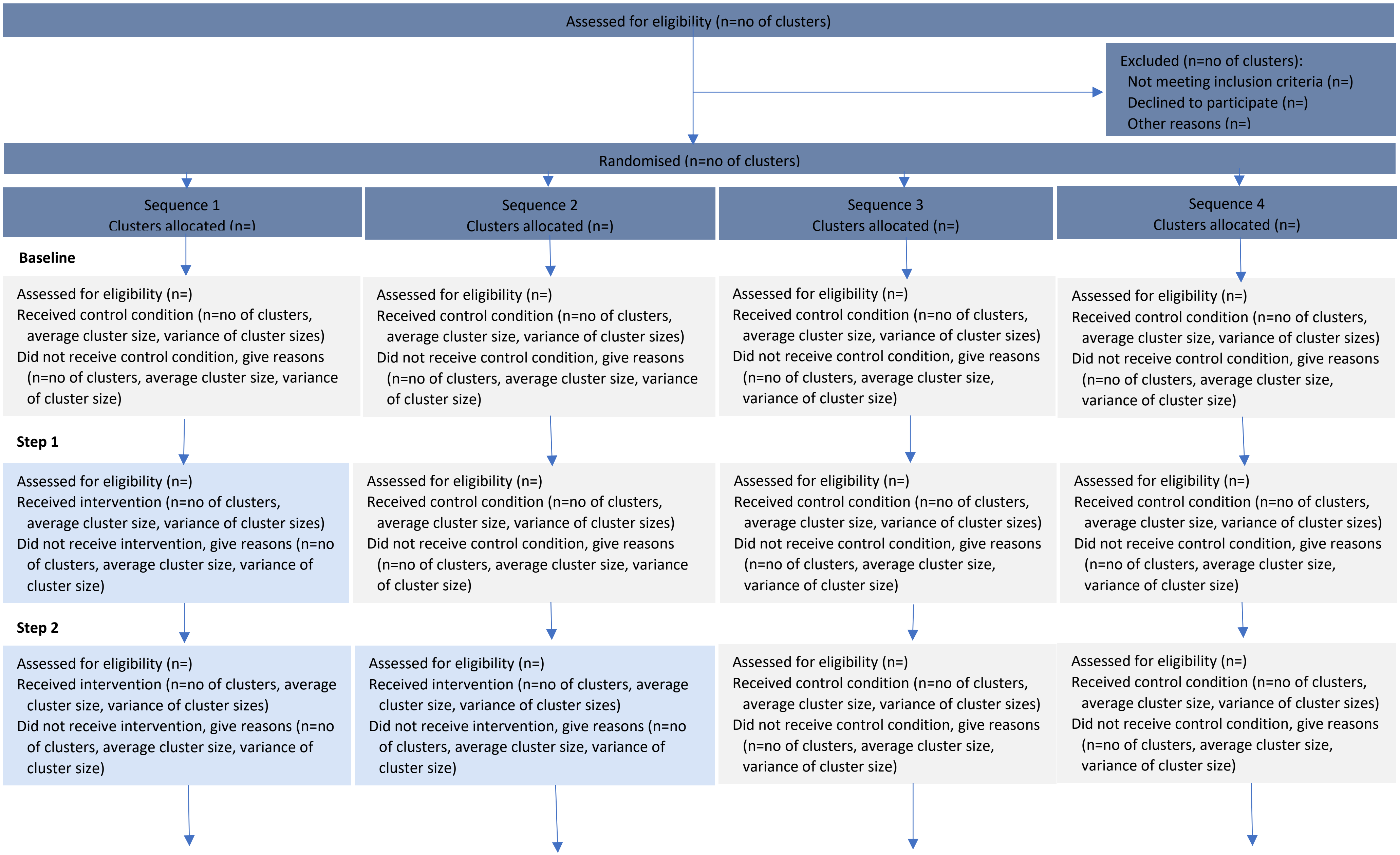
Assessed for eligibility (n=no of clusters)
Excluded (n=no of clusters):
Not meeting inclusion criteria (n=)
Declined to participate (n=)
Other reasons (n=)
Randomised (n=no of clusters)
Sequence 1
Clusters allocated (n=)
Sequence 2
Clusters allocated (n=)
Sequence 3
Clusters allocated (n=)
Sequence 4
Clusters allocated (n=)
Baseline
Assessed for eligibility (n=)
Received control condition (n=no of clusters, average cluster size, variance of cluster sizes)
Did not receive control condition, give reasons (n=no of clusters, average cluster size, variance of cluster size)
Assessed for eligibility (n=)
Received control condition (n=no of clusters, average cluster size, variance of cluster sizes)
Did not receive control condition, give reasons (n=no of clusters, average cluster size, variance of cluster size)
Assessed for eligibility (n=)
Received control condition (n=no of clusters, average cluster size, variance of cluster sizes)
Did not receive control condition, give reasons (n=no of clusters, average cluster size, variance of cluster size)
Assessed for eligibility (n=)
Received control condition (n=no of clusters, average cluster size, variance of cluster sizes)
Did not receive control condition, give reasons (n=no of clusters, average cluster size, variance of cluster size)
Step 1
Assessed for eligibility (n=)
Received intervention (n=no of clusters, average cluster size, variance of cluster sizes)
Did not receive intervention, give reasons (n=no of clusters, average cluster size, variance of cluster size)
Assessed for eligibility (n=)
Received control condition (n=no of clusters, average cluster size, variance of cluster sizes)
Did not receive control condition, give reasons (n=no of clusters, average cluster size, variance of cluster size)
Assessed for eligibility (n=)
Received control condition (n=no of clusters, average cluster size, variance of cluster sizes)
Did not receive control condition, give reasons (n=no of clusters, average cluster size, variance of cluster size)
Assessed for eligibility (n=)
Received control condition (n=no of clusters, average cluster size, variance of cluster sizes)
Did not receive control condition, give reasons (n=no of clusters, average cluster size, variance of cluster size)
Step 2
Assessed for eligibility (n=)
Received intervention (n=no of clusters, average cluster size, variance of cluster sizes)
Did not receive intervention, give reasons (n=no of clusters, average cluster size, variance of cluster size)
Assessed for eligibility (n=)
Received intervention (n=no of clusters, average cluster size, variance of cluster sizes)
Did not receive intervention, give reasons (n=no of clusters, average cluster size, variance of cluster size)
Assessed for eligibility (n=)
Received control condition (n=no of clusters, average cluster size, variance of cluster sizes)
Did not receive control condition, give reasons (n=no of clusters, average cluster size, variance of cluster size)
Assessed for eligibility (n=)
Received control condition (n=no of clusters, average cluster size, variance of cluster sizes)
Did not receive control condition, give reasons (n=no of clusters, average cluster size, variance of cluster size)

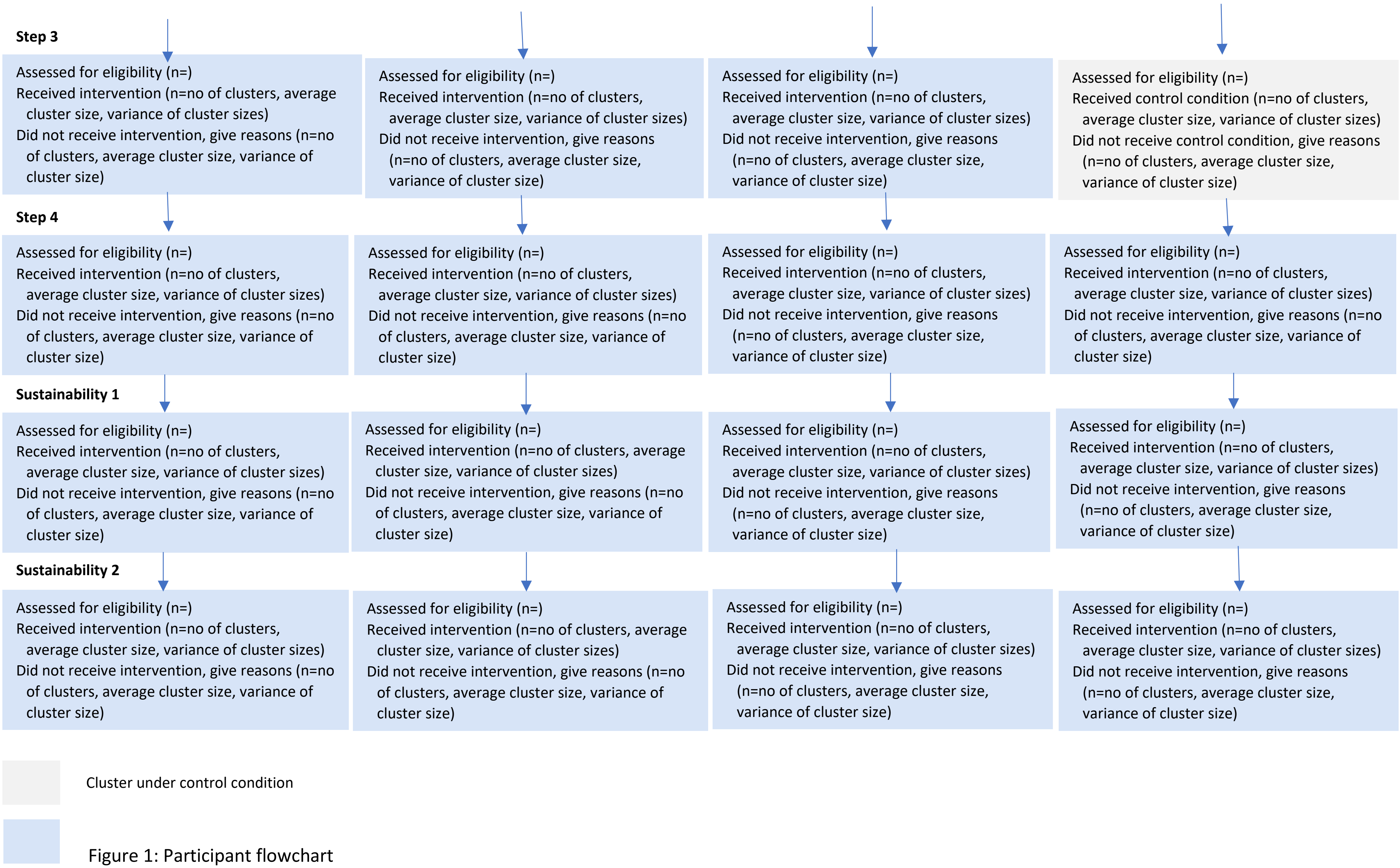

Figure 1: Participant flowchart

Table 1 Baseline characteristics

	**Control condition** Frequency (%) or Median (IQR) n=xx	**Intervention condition** Frequency (%) or Median (IQR) n=xx
DIVA tool outcome		
- DIVA	xx (xx%)	xx (xx%)
- Potential DIVA	xx (xx%)	xx (xx%)
- Non-DIVA	xx (xx%)	xx (xx%)
Sex		
- Male	xx (xx%)	xx (xx%)
Age in years	xx (xx-xx)	xx (xx-xx)
Bodyweight		
- Obese	xx (xx%)	xx (xx%)
- Overweight	xx (xx%)	xx (xx%)
- Normal weight	xx (xx%)	xx (xx%)
- Underweight	xx (xx%)	xx (xx%)
Number of comorbidities		
- 0	xx (xx%)	xx (xx%)
- 1	xx (xx%)	xx (xx%)
- 2	xx (xx%)	xx (xx%)
- 3	xx (xx%)	xx (xx%)
- >3	xx (xx%)	xx (xx%)
Skin integrity		
- Good	xx (xx%)	xx (xx%)
- Fair	xx (xx%)	xx (xx%)
- Poor	xx (xx%)	xx (xx%)
Skin type		
- Pale white	xx (xx%)	xx (xx%)
- White	xx (xx%)	xx (xx%)
- Moderate brown	xx (xx%)	xx (xx%)
- Dark brown	xx (xx%)	xx (xx%)
- Deeply pigmented dark	xx (xx%)	xx (xx%)
Type of admission		
- Medical (emergent)	xx (xx%)	xx (xx%)
- Medical (elective)	xx (xx%)	xx (xx%)
- Surgical (emergent)	xx (xx%)	xx (xx%)
- Surgical (elective)	xx (xx%)	xx (xx%)
- Trauma	xx (xx%)	xx (xx%)
- Oncology/haematology	xx (xx%)	xx (xx%)
- Other	xx (xx%)	xx (xx%)
Patient status		
- Diaphoretic (sweaty)	xx (xx%)	xx (xx%)
- Unconscious/non-communicative	xx (xx%)	xx (xx%)
- Restless/agitated	xx (xx%)	xx (xx%)
- None of the above	xx (xx%)	xx (xx%)
Dominant side insertion		
- Yes	xx (xx%)	xx (xx%)
PIVC inserted by		
- Vascular Access Specialist	xx (xx%)	xx (xx%)
- Nurse	xx (xx%)	xx (xx%)

- Medical officer	xx (xx%)	xx (xx%)
- Other	xx (xx%)	xx (xx%)
Reason for PIVC insertion		
- Fluids	xx (xx%)	xx (xx%)
- Medication	xx (xx%)	xx (xx%)
- Blood products	xx (xx%)	xx (xx%)
- Prophylactic insertion	xx (xx%)	xx (xx%)
- Blood sampling	xx (xx%)	xx (xx%)
- Other	xx (xx%)	xx (xx%)
Insertion site		
- Anterior upper forearm	xx (xx%)	xx (xx%)
- Posterior lower forearm	xx (xx%)	xx (xx%)
- Wrist	xx (xx%)	xx (xx%)
- Posterior upper forearm	xx (xx%)	xx (xx%)
- Hand	xx (xx%)	xx (xx%)
- Cubital fossa	xx (xx%)	xx (xx%)
- Anterior lower forearm anterior upper arm	xx (xx%)	xx (xx%)
- Posterior upper arm	xx (xx%)	xx (xx%)
Intravenous cannula size		
- 22G	xx (xx%)	xx (xx%)
- 20G	xx (xx%)	xx (xx%)
- 18G	xx (xx%)	xx (xx%)
10-15cm extension tubing		
- Yes	xx (xx%)	xx (xx%)
3-way connector		
- Yes	xx (xx%)	xx (xx%)
Infection at recruitment		
- Yes	xx (xx%)	xx (xx%)
Pre-existing wound		
- Yes	xx (xx%)	xx (xx%)
Dressings		
- Simple transparent dressing (i.e., no foam or fabric border)	xx (xx%)	xx (xx%)
- Bordered transparent dressing	xx (xx%)	xx (xx%)
- Integrated securement dressing (e.g., SorbaView)	xx (xx%)	xx (xx%)
- Fabric dressing (e.g., Primapore)	xx (xx%)	xx (xx%)
- Unknown	xx (xx%)	xx (xx%)
- Other	xx (xx%)	xx (xx%)
Securements		
- No securements	xx (xx%)	xx (xx%)
- Non-sterile tape	xx (xx%)	xx (xx%)
- Sterile tape	xx (xx%)	xx (xx%)
- Tubular bandage (e.g., Tubifast)	xx (xx%)	xx (xx%)
- Bandage	xx (xx%)	xx (xx%)

- Sutureless Securement Device	xx (xx%)	xx (xx%)
- Hyperfix / Mefix /	xx (xx%)	xx (xx%)
Fixomull	xx (xx%)	xx (xx%)
- Tissue adhesive	xx (xx%)	xx (xx%)
- Armboard / splint	xx (xx%)	xx (xx%)
- Other		
Intravenous antibiotics during PIVC dwell		
- Yes	xx (xx%)	xx (xx%)
Previous PIVC inserted during admission		
- Yes	xx (xx%)	xx (xx%)
Other indwelling vascular devices		
- Central venous catheter/peripherally inserted central-line catheter	xx (xx%)	xx (xx%)
- Midline	xx (xx%)	xx (xx%)
- Port	xx (xx%)	xx (xx%)
Other indwelling devices		
- Wound drain	xx (xx%)	xx (xx%)
- Stoma	xx (xx%)	xx (xx%)
- Tracheostomy	xx (xx%)	xx (xx%)

IQR: interquartile range; DIVA: difficult intravenous access; PIVC: peripheral intravenous catheter.
Data are missing for xx.

Table 2: Associated between study phase and study outcomes (adjusted for calendar month)

	**Baseline**	**Implementation**	**Sustainability**	**Baseline vs Implementation**		**Implementation vs Sustainability**	
**Outcome**	N (%) or Median (IQR)	N (%) or Median (IQR)	N (%) or Median (IQR)	Effect estimate (95% CI)	*P*-value	Effect estimate (95% CI)	*P*-value
First attempt insertion success among DIVA patients	xx (xx%)	xx (xx%)	xx (xx%)	x.x (x.x to x.x)[1]	0.xx	x.x (x.x to x.x)[1]	0.xx
First attempt insertion success among all patients	xx (xx%)	xx (xx%)	xx (xx%)	x.x (x.x to x.x)[1]	0.xx	x.x (x.x to x.x)[1]	0.xx
Ultrasound adoption at first attempt among DIVA patients	xx (xx%)	xx (xx%)	xx (xx%)	x.x (x.x to x.x)[1]	0.xx	x.x (x.x to x.x)[1]	0.xx
Ultrasound adoption at any attempt among DIVA patients	xx (xx%)	xx (xx%)	xx (xx%)	x.x (x.x to x.x)[1]	0.xx	x.x (x.x to x.x)[1]	0.xx
Number of PIVC insertion attempts	xx (xx to xx)	xx (xx to xx)	xx (xx to xx)	x.x (x.x to x.x)[2]	0.xx	x.x (x.x to x.x)[2]	0.xx
Successful PIVC placement	xx (xx%)	xx (xx%)	xx (xx%)	x.x (x.x to x.x)[1]	0.xx	x.x (x.x to x.x)[1]	0.xx
Time to therapy	xx (xx to xx)	xx (xx to xx)	xx (xx to xx)	x.x (x.x to x.x)[3]	0.xx	x.x (x.x to x.x)[3]	0.xx
Alternate device use	xx (xx%)	xx (xx%)	xx (xx%)	x.x (x.x to x.x)[1]	0.xx	x.x (x.x to x.x)[1]	0.xx
Alternate route use	xx (xx%)	xx (xx%)	xx (xx%)	x.x (x.x to x.x)[1]	0.xx	x.x (x.x to x.x)[1]	0.xx
Use of unnecessary PIVCs	xx (xx%)	xx (xx%)	x (xx%)	x.x (x.x to x.x)[1]	0.xx	x.x (x.x to x.x)[1]	0.xx
Time to post-insertion PIVC failure	xx (xx%)	xx (xx%)	xx (xx%)	x.x (x.x to x.x)[3]	0.xx	x.x (x.x to x.x)[3]	0.xx

PIVC dwell time	xx (xx to xx)	xx (xx to xx)	xx (xx to xx)	x.x (x.x to x.x)[3]	0.xx	x.x (x.x to x.x)[3]	0.xx
Insertion and post-insertion complications	xx (xx%)	xx (xx%)	xx (xx%)	x.x (x.x to x.x)[1]	0.xx	x.x (x.x to x.x)[1]	0.xx
Patient/carer satisfaction with insertion procedure	xx (xx to xx)	xx (xx to xx)	xx (xx to xx)	x.x (x.x to x.x)[2]	0.xx	x.x (x.x to x.x)[2]	0.xx
Patient/carer pain with insertion procedure	xx (xx to xx)	xx (xx to xx)	xx (xx to xx)	x.x (x.x to x.x)[2]	0.xx	x.x (x.x to x.x)[2]	0.xx
Staff satisfaction with insertion procedure	xx (xx to xx)	xx (xx to xx)	xx (xx to xx)	x.x (x.x to x.x)[2]	0.xx	x.x (x.x to x.x)[2]	0.xx
Primary BSI and S. Aureus BSI	xx (xx%)	xx (xx%)	xx (xx%)	x.x (x.x to x.x)[1]	0.xx	x.x (x.x to x.x)[1]	0.xx

IQR: interquartile range; CI: confidence interval; DIVA: difficult intravenous access; PIVC: peripheral intravenous catheter; BSI: blood stream infection.
[1]Odds ratio; [2]correlation coefficient; [3]hazard ratio.
Data are missing for xx.
