# Supplementary material for: Improving difficult peripheral intravenous access requires thought, training and technology (DART3): a stepped-wedge, cluster randomised controlled trial protocol
Source: BMC Health Serv Res. 2023 Jun 7;23:587. doi: 10.1186/s12913-023-09499-0 (PMC10249237; doi:10.1186/s12913-023-09499-0)
Supplement: Supplementary file 4 — Supplementary Material 4 [file 12913_2023_9499_MOESM4_ESM.docx]

**Supplementary material 4. DART^3^ Co-design implementation strategies to support DIVA tool roll out**

| **Intervention components** | **Subcomponents** | **Change principles underpinning each subcomponent** |
| --- | --- | --- |
| DIVA risk assessment and escalation pathway co-development | 1. advisory group 2. summary of systematic reviews to encourage exchange of research findings 3. summary of qualitative findings from stakeholder interviews 4. identification of key principles 5. local health care context adaption 6. instrument piloting at sites | - engages sites in instrument co-development and adaption - provides a clear rationale for change - develops agreement regarding concrete and specific change goals including study objectives and priorities - ensures the instrument is tailored to focus on the site’s priorities in health care context - determines the reliability of the instrument |
| Education and ultrasound training program | 1. Co-development of ultrasound educational materials 2. Ultrasound training pathway 3. develop plans for access to resources and self-education opportunity | - provides an educational framework to support staff delivering the intervention - recognises the training needs of participants - includes interactive experiences with the focused on shared learning and problem-solving - uses credible and experienced trainers (Chief investigator’s) - promotes access to resources and provides opportunities for practice |
| Communication strategies | 1. Identification of strategies which support broad implementation. 2. Mid intervention feedback | - addresses systems, operations, structures and workforce issues that may impact intervention being delivered as planned e.g., lanyards, DIVA t-shirts, equipment checks - engages sites in owning and driving the program - monitors and provides feedback about the implementation and change process during the intervention and study. |
| Site and central support for study | 1. feedback forum 2. Dart3 newsletter 3. access to online learning resources 4. quarterly email endorsed by key stakeholders 5. study champions to lead organisational change 6. resources for improving sites use of research and DIVA instrument | - provides feedback about current practice - Engages sites and study timeline and proceedings - uses champions to model and promote the use of the intervention - provides feedback about any changes to the intervention recognises the expertise of the participants - promotes ongoing interaction of the sites with the study |
