# Supplementary material for: Improving difficult peripheral intravenous access requires thought, training and technology (DART3): a stepped-wedge, cluster randomised controlled trial protocol
Source: BMC Health Serv Res. 2023 Jun 7;23:587. doi: 10.1186/s12913-023-09499-0 (PMC10249237; doi:10.1186/s12913-023-09499-0)
Supplement: Supplementary file 5 — Supplementary Material 5 [file 12913_2023_9499_MOESM5_ESM.docx]

| **Category** | **Data Elements** |
| --- | --- |
| Person data | Demographic information, patient skin type, skin integrity, current infection status, type of infection, patient comorbidity, PIVC history, device/implantable in situ |
| Device data | Study phase; details of PIVC insertion (including reason and time of PIVC order, time of PIVC insertion, number of attempts, staff involved in PIVC insertion, ultrasound use for PIVC attempts); patient vein quality assessment by PIVC inserters; level of satisfaction with PIVC insertion pathway; details of PIVC device (including PIVC location, vein site, type of PIVC, size, length, dressing, securement insitu); patient satisfaction and pain; alternative access if PIVC failed; treatment outcome; insertion complications |
| Daily check | Number of intravascular device; PIVC dressing status; Complications at PIVC site (including pain, erythema, swelling, bruising, warmth, purulence and dislodgement) |
| Device outcome | Details of PIVC removal (time, reason, complications followed up to 24hrs post-removal); Study PIVC site assessment; Use of PIVC device (primary use, time of first and last use) |
| Patient outcome | Patient outcome; Adverse event 48hrs post-removal; Pathology results (including PIVC swabs, blood culture reports) |

**Supplementary material 5. DART^3^ data elements.**
