# Supplementary material for: Improving difficult peripheral intravenous access requires thought, training and technology (DART3): a stepped-wedge, cluster randomised controlled trial protocol
Source: BMC Health Serv Res. 2023 Jun 7;23:587. doi: 10.1186/s12913-023-09499-0 (PMC10249237; doi:10.1186/s12913-023-09499-0)
Supplement: Supplementary file 6 — Supplementary Material 6 [file 12913_2023_9499_MOESM6_ESM.docx]

**Supplementary material 6. DART^3^ Process evaluation domains, research questions and data collection**

| **Process evaluation domains** | **Research questions** | **Core information sought** | **Data type and source** | **Data management** |
| --- | --- | --- | --- | --- |
| Domain 1: Implementation | 1. How was intervention implemented at each site including: 2. what components were delivered? 3. to what extent were the *essential elements* or key principles implemented? | Intervention components used and how sites ask that they be adapted or tailored.  Delivery of the intervention at each site including instrument components, delivery format, participant recruitment and consent, educational materials, the fidelity with which essential elements were adhered to, any changes to the implementation plan and or follow-up activities. | Initial Site visits and observations  Feedback via in person and/or telephone discussion with research staff and local champions  Final site visits – semi structured interviews see Domain 3.  DART 3 Trial records and RedCap  To undertake interviews with a purposive sample of staff involved in intervention and intervention implementation to ascertain guideline acceptability, how workflow supported what do not support | Collated spreadsheet data  Checklists  Observation (semi structured)  Field notes |
| **Additional information regarding our framework for documenting intervention implementation is outlined in Table 3** | | | | |
| Domain 2: Participation and response | 1. How did staff understand, respond and participate with the intervention over time and across different sites? What were their levels of satisfaction and participation and what was the acceptability of the intervention? 2. Were there any effects not captured by outcome measures that the intervention did not have including unexpected effects? | Advisory group participation and responses including roles of attendees, proportion of interviewees who attended advisory groups and the nature of participation for example the type and extent of their interaction such as the ABC insertion or study recruitment.  Staff evaluation of ultrasound and study intervention training sessions  How did staff and study participants and the overall health care system responds the intervention including any unexpected effects | Minutes of advisory group meetings  Semi structured interviews and observation  Self-reported evaluation and feedback  informal conversations during study period and after training sessions  Meetings and other informal conversations | Electronic records  Completed feedback forms  Field notes  Audio recordings, transcriptions or fieldnotes/memos from interviews  Checklists  Observation (semi structured) |
| Domain 3: Context | 1. Determine what factors including managerial, economic, organisational and work level that affected implementation of the intervention. | Initial site visits to obtain information on context and usual practice collected through interviews with staff involved in the implementation and delivery of the intervention, using purposive sampling to obtain a range of participants (Box A).  In person and/or telephone interviews with research staff and local champions in the intervention phase to obtain information regarding the implementation process, acceptability of the intervention, barriers and clinical decisions affecting the use of the intervention.  Final site visits to undertake interviews with a purposive sample of staff involved in implementation or intervention delivery to ascertain guideline acceptability, how workflows supported (or did not support) the pathways’ implementation, what was helpful or challenging when operationalising the pathways and whether they were useful in practice (see Box A for Interview guide). | | Audio recordings, transcriptions or fieldnotes/memos from interviews  Spreadsheet of interview responses |
| Domain 4: Application of theory | 1. Determine how the application of the COM- B framework impacted the implementation of the intervention | | | Narrative synthesis of above data |
| Across domains | 6. How might the relationships between the intervention, implementation plan, the people and the context in each site have shaped the variations in these effects?  7. What lessons can we learn from this study that might be relevant for future implementation projects at these sites in future | | | Narrative synthesis of above data |
